# A Pimarane Diterpene and Cytotoxic Angucyclines from a Marine-Derived *Micromonospora* sp. in Vietnam’s East Sea

**DOI:** 10.3390/md13095815

**Published:** 2015-09-15

**Authors:** Michael W. Mullowney, Eoghainín Ó hAinmhire, Urszula Tanouye, Joanna E. Burdette, Van Cuong Pham, Brian T. Murphy

**Affiliations:** 1 Department of Medicinal Chemistry and Pharmacognosy, College of Pharmacy, University of Illinois at Chicago, 833 South Wood Street (MC 781), Room 539, Chicago, IL 60612-7231, USA; E-Mails: mmullo2@uic.edu (M.W.M); eohain2@uic.edu (E.Ó.); urszula.tanouye@gmail.com (U.T.); joannab@uic.edu (J.E.B.); 2 Center for Biomolecular Sciences, College of Pharmacy, University of Illinois at Chicago, Molecular Biology Research Building, 900 South Ashland Avenue (MC 870), Room 3150, Chicago, IL 60607-7173, USA; 3 Institute of Marine Biochemistry, Vietnam Academy of Science and Technology, 18 Hoàng Quốc Việt, Cầu Giấy, Hà Nội 10000, Vietnam; E-Mail: phamvc@yahoo.com

**Keywords:** actinomycete, marine, *Micromonospora*, ovarian cancer, Vietnam, diterpene

## Abstract

A screening of our actinomycete fraction library against the NCI-60 SKOV3 human tumor cell line led to the isolation of isopimara-2-one-3-ol-8,15-diene (**1**), lagumycin B (**2**), dehydrorabelomycin (**3**), phenanthroviridone (**4**), and WS-5995 A (**5**). These secondary metabolites were produced by a *Micromonospora* sp. isolated from sediment collected off the Cát Bà peninsula in the East Sea of Vietnam. Compound **1** is a novel Δ^8,9^-pimarane diterpene, representing one of approximately 20 actinomycete-produced diterpenes reported to date, while compound **2** is an angucycline antibiotic that has yet to receive formal characterization. The structures of **1** and **2** were elucidated by combined NMR and MS analysis and the absolute configuration of **1** was assigned by analysis of NOESY NMR and CD spectroscopic data. Compounds **2**–**5** exhibited varying degrees of cytotoxicity against a panel of cancerous and non-cancerous cell lines. Overall, this study highlights our collaborative efforts to discover novel biologically active molecules from the large, underexplored, and biodiversity-rich waters of Vietnam’s East Sea.

## 1. Introduction

Natural products and their synthetic derivatives are essential toward the discovery of drugs, accounting for greater than 74% of marketed small molecule therapies [1,2]. In particular, actinomycete bacteria have provided us with an abundance of bioactive compounds, including more than half of marketed antibiotics and many clinically useful anticancer drugs [2]. Of the different classes of molecules produced by actinomycetes, the terpenes are a structurally diverse group of natural products with approximately 60,000 members. A recent study unveiled that the capacity of actinomycetes to produce diterpenes has been significantly underestimated, where 25 of 100 randomly selected strains were identified as potential diterpene producers [3]. Despite this, only an estimated twenty diterpenes of actinomycete origin have been reported to date [4]. This is an exceedingly scarce fraction of the nearly 12,000 diterpenes described in the peer-reviewed literature, which are predominantly plant- and fungal-derived. In the current study, we report the rare isolation of a diterpene from an actinomycete strain.

**Figure 1:**
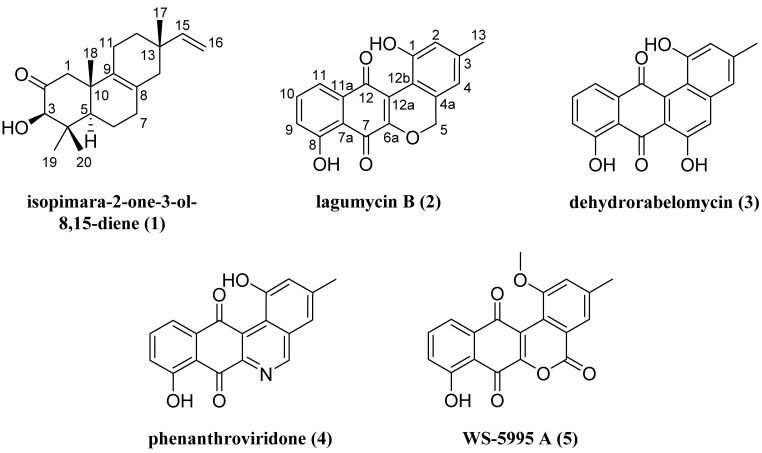
Structures of compounds **1**–**5**.

Our program explores the potential of marine and fresh water-derived actinomycete secondary metabolites to serve as antibiotic and anticancer drug leads [5,6]. As part of this program, the University of Illinois at Chicago has partnered with the Vietnam Academy of Science and Technology to explore the potential of Vietnam’s East Sea to provide such leads. The East Sea in Vietnam covers an area of approximately three-million km^2^ and traces 3000 km of coastline. This stretch is comprised of a multitude of microenvironments covering depths from 200 m to 5000 m. The marine biodiversity within the East Sea is considered to be some of the most extensive in the world, yet it remains poorly understood and explored. In the current study, through an *in vitro* growth inhibition screening of our fraction library against the NCI-60 SKOV3 human tumor cell line, we identified an actinomycete isolated from sediment collected off the Cát Bà peninsula in the East Sea of Vietnam. From this strain we isolated and characterized the novel diterpene isopimara-2-one-3-ol-8,15-diene (**1**) and lagumycin B (**2**), an angucycline that has yet to receive full structural characterization in the peer-reviewed literature (Figure 1) [7]. In addition, we identified the previously reported angucyclines dehydrorabelomycin (**3**) [8,9], phenanthroviridone (**4**) [10,11], and WS-5995 A (**5**) [12,13]. Herein we present the structure elucidation and biological activity of these compounds.

## 2. Results and Discussion

### 2.1. Structure Elucidation of Isopimara-2-one-3-ol-8,15-diene (**1**) and Lagumycin B (**2**)

Following a series of chromatographic experiments, **1** was obtained as a colorless solid. Combined HRMS and NMR experiments allowed for the assignment of the molecular formula as C_20_H_30_O_2_, indicative of six degrees of unsaturation. ^1^H and ^13^C NMR data are shown in Table 1. In the ^1^H NMR spectrum, we observed evidence for four sp^3^ quaternary carbon-bound methyl groups, one vinyl group, an oxymethine geminal to a hydroxy group, a pair of geminal hydrogens adjacent to a carbonyl, and additional methylene and methine groups on **1** that constituted the remainder of a pimarane diterpene core skeleton.

**Table 1:** ^1^H and ^13^C NMR data (CDCl_3_) of **1**.

Position	^13^C, Type *^a^*	^1^H, Mult. (*J*, Hz) *^b,c^*
1_ax_	49.8, CH_2_	2.25, d (12.3)
1_eq_		2.58, d (12.3)
2	211.8,C	
3	83.0, CH	3.91, d (4.0)
3-OH		3.44, d (4.0)
4	45.3, C	
5	50.3, CH	1.78, m
6	18.8, CH_2_	1.58, m
		1.80, m
7	32.3, CH_2_	2.03, m
8	126.4, C	
9	134.3, C	
10	43.8, C	
11_ax_	21.4, CH_2_	1.76, m
11_eq_		1.88, m
12_eq_	34.8, CH_2_	1.33, m
12_ax_		1.53, m
13	35.3, C	
14	41.6, CH_2_	1.76, m
		1.88, m
15	145.9, CH	5.72, dd (17.5, 10.7)
16	111.2, CH_2_	4.85, dd (17.5, 1.4)
		4.92, dd (10.7, 1.4)
17	28.3, CH_3_	0.98, s
18	20.6, CH_3_	0.93, s
19	29.3, CH_3_	1.21, s
20	16.5, CH_3_	0.72, s

*^a^* 226.2 MHz; *^b^* 600 MHz; *^c^* s = singlet; d = doublet; dd = doublet of doublets; m = multiplet.

**Figure 2:**
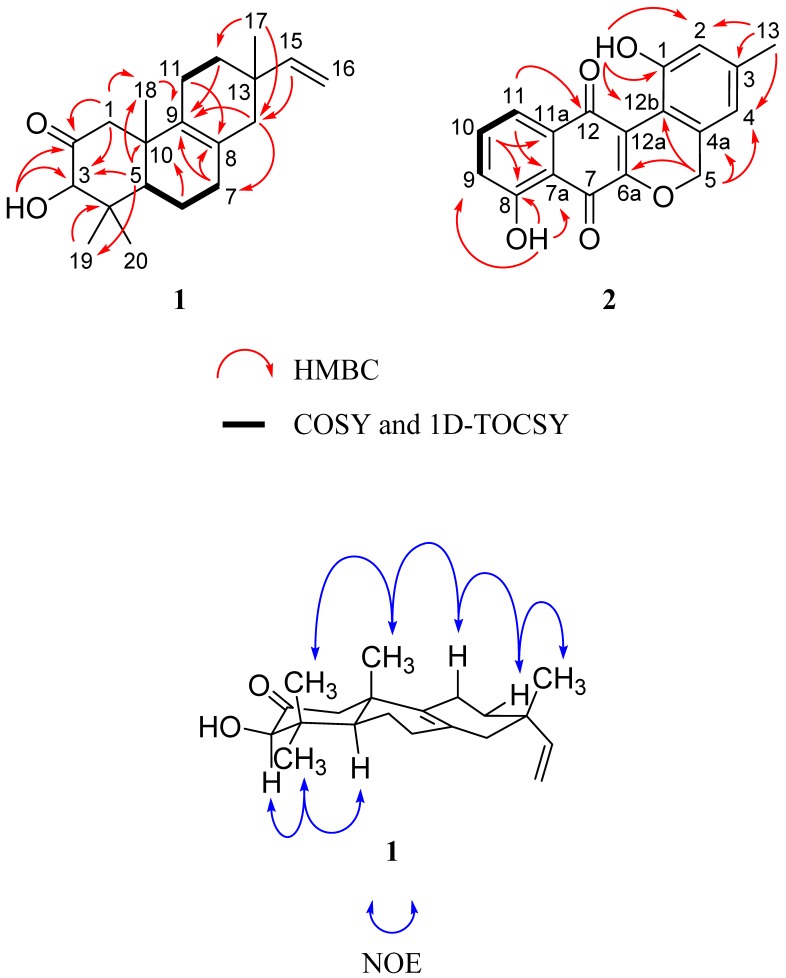
Key 2D NMR correlations of **1** and **2**.

Analysis of ^13^C- DEPTQ and HSQC NMR data for **1** indicated the presence of one carbonyl carbon (δ_C_ 211.8, C-2), two fully-substituted endocyclic alkene carbons (δ_C_ 126.4, C-8; 134.3, C-9), one vinyl methylene carbon (δ_C_ 111.2, C-16), one vinyl methine carbon (δ_C_ 145.9, C-15), one oxymethine carbon (δ_C_ 83.0, C-3), and fourteen additional sp^3^ carbons. Given that the molecular formula of **1** afforded six degrees of unsaturation and the molecule contained one carbonyl, one vinyl, and one endocyclic alkene group, the remaining three degrees were satisfied by the tricyclic pimarane ring system. Key HMBC, COSY, and 1D-TOCSY correlations are given in Figure 2.

The connectivity of the two distinct spin systems of C-11–C-12 and C-5–C-6–C-7 was determined by interpretation of the COSY NMR spectrum. Confirmation of these assignments was supported by a series of 1D-TOCSY experiments that exploited the solvent effect of C_6_D_6_ on lower frequency chemical shifts, which served to deconvolute the signal overlap observed when ^1^H NMR experiments were run in CDCl_3_ (Table S2 and Figures S5–S8) [14]. At this point in the elucidation process, we used HMBC correlations of methyl groups in **1** to piece together the tricyclic diterpene core. Geminal methyl groups were confirmed at C-4 based on HMBC correlations from H_3_-19 and H_3_-20 to C-3, C-4, and C-5. Signal H_3_-18 exhibited correlations to C-1, C-9, and C-10, suggesting a bond to the quaternary C-10. The connectivity of ring A was further established by observation of HMBC correlations from H_2_-1 to C-3, C-5, C-9, C-10, and C-18, suggesting that this methylene was between the C-2 carbonyl and C-10. Additionally, the oxymethine signal (H-3) showed HMBC couplings to C-2, C-4, C-19, and C-20, while the hydroxy signal (3-OH) displayed correlations to C-2 and C-3. This supported that H-3 and 3-OH were connected to the same carbon, which was positioned between the carbonyl at C-2 and the quaternary C-4. Couplings were also observed from H-5 to C-4 and C-10, solidifying the fusion of rings A and B. The connectivity of the C-5–C-6–C-7 spin system from ring A to the remainder of the core was established by observation of HMBC correlations from H_2_-7 to C-8 and C-9. Correlations from H_2_-14 to C-7, C-8, C-9, C-12, C-15, and C-17 placed it between the alkene C-8 and quaternary C-13. Resonance H_3_-17 showed HMBC correlations to C-12, C-13, C-14, and C-15, placing it at position 13. HMBC correlations between the vinyl methine hydrogen (H-15) and C-13, C-14, and C-17 supported this, while correlations from H_2_-16 to C-15 and C-13 provided further evidence for connectivity of the vinyl group to C-13. The remaining connectivity of ring C was satisfied by HMBC correlations from H_2_-11 to C-8, C-9, and C-13.

A 2D-NOESY NMR experiment was employed in order to determine the relative stereochemistry of **1**, revealing correlations between H_3_-20 and H_3_-18, H_3_-18 and H-11_ax_, H-11_ax_ and H-12_eq_, and H-12_eq_ and H_3_-17; this suggested that these hydrogens projected out from the same face of the molecule. This orientation was supported by the observation of NOESY correlations between hydrogens H-3 and H_3_-19, and H_3_-19 and H-5, projecting from the opposite face of the molecule (Figure 2).

To identify the absolute configuration of centers in **1**, a CD spectrum was acquired and analyzed (Figure S11). Compound **1** exhibited a positive Cotton effect at the *n* → π* carbonyl transition of 290 nm, indicating that the β-axial methyl group (C-18) was in the rear upper left octant when the octant rule was applied [15]. Thus, the absolute configuration of stereocenters in **1** was elucidated as 3*R*, 5*R*, 10*S*, and 13*S*, establishing the structure of **1** as shown.

Following a series of chromatographic steps, **2** was obtained as a pale orange solid. Combined HRMS and NMR experiments allowed for the assignment of the molecular formula as C_18_H_12_O_5_, indicative of thirteen degrees of unsaturation. The UV absorption profile (maxima of 204, 280, 310, and 410 nm) of **2** displayed characteristics of an extended aromatic ring system similar to the previously reported angucycline isolates **3**, **4**, and **5**. ^1^H and ^13^C NMR data are shown in Table 2. A singlet resonance at δ_H_ 5.19 (2H, H-5) observed in the ^1^H NMR spectrum indicated the presence of oxygenated methylene protons. Two highly deshielded resonances at δ_H_ 10.45 (1H, 1-OH) and δ_H_ 11.70 (1H, 8-OH) gave evidence of two hydroxy peri-protons. The observation of three aromatic signals at δ_H_ 7.30 (1H, H-9), δ_H_ 7.81 (1H, H-11), and δ_H_ 7.66 (1H, H-10) indicated the presence of a tri-substituted aromatic ring. Additionally, two singlet resonances were observed in the aromatic region at δ_H_ 6.54 (1H, H-4) and δ_H_ 6.85 (1H, H-2), indicating the presence of isolated hydrogens on a separate, tetrasubstituted aromatic system. Finally, a singlet resonance at δ_H_ 2.33 (3H, H_3_-13) evidenced one aromate-bound methyl group.

Analysis of ^13^C-DEPTQ NMR data suggested the presence of two quinone carbonyls (δ_C_ 186.6, C-12; 183.4, C-7), two hydroxy-substituted aromatic carbons (161.7, C-8; 154.5, C-1), one oxygenated methylene carbon (δ_C_ 70.7, C-5), one methyl carbon (δ_C_ 21.3, H_3_-13), and an additional twelve aromatic carbons. Given that the molecular formula afforded thirteen degrees of unsaturation and the molecule contained two carbonyls and seven double bonds, the remaining degrees were satisfied by the fused angucycline ring system.

Interpretation of COSY NMR data defined one distinct aromatic spin system that connected C-9–C-10–C-11, supporting the aforementioned evidence for a 1,2,3-trisubstitued benzene moiety. A phenolic hydroxy peri-proton at δ_H_ 11.70 showed HMBC correlations to C-7a, C-8, and C-9, establishing hydroxy connectivity to C-8. An aromatic hydrogen at H-10 exhibited an HMBC correlation to C-11a, completing assignments for the 1,2,3-trisubstitued benzene. An HMBC correlation observed between aromatic hydrogen H-11 and the carbonyl C-12 was critical to establishment of the phenol position relative to the central quinone ring. The positions of the C-7 and C-12 relative to the oxygenated carbon at C-6a were based on comparison to previously reported ^13^C chemical shift values of similarly substituted paraquinones [16,17]. The 1-OH peri-proton showed HMBC correlations to C-1, C-12b, and C-2, providing evidence for connectivity of the hydroxy group to C-1. The singlet aromatic methyl signal at H_3_-13 exhibited correlations to C-3, C-2, and C-4 in the HMBC spectrum, placing it *meta* to the hydroxy group at C-1. HMBC correlations from the oxygenated methylene at H_2_-5 to C-4a, C-4, C-12b, and C-6a established connectivity of the naphthoquinone fragment to the phenol moiety. Key COSY and HMBC correlations are given in Figure 2. Thus, the structure of **2** is as shown with the name lagumycin B, following the structure and nomenclature established in the 1995 dissertation by Balk-Bindseil and Laatsch [7].

The characterization of known compounds **3**–**5** was based on comparison of ^1^H NMR and HRMS data with those appearing in literature (Figures S18–S23) [8,9,10,11,12,13].

**Table 2:** ^1^H and ^13^C NMR data (CDCl_3_) of **2**.

Position	^13^C, Type *^a^*	^1^H, Mult. (*J*, Hz) *^b,c^*
1	154.5, C	
1-OH		10.45, s
2	121.1, CH	6.85, s
3	143.7, C	
4	118.2, CH	6.54, s
4a	130.3, C	
5	70.7, CH_2_	5.19, s
6a	157.0, C	
7	183.4, C	
7a	113.5, C	
8	161.7, C	
8-OH		11.70, s
9	125.2, CH	7.30, d (8.1)
10	137.2, CH	7.66, t (8.1)
11	121.3, CH	7.81, d (8.1)
11a	132.2, C	
12	186.6, C	
12a	124.4, C	
12b	109.9, C	
13	21.3, CH_3_	2.33, s

*^a^* 226.2 MHz; *^b^* 600 MHz; *^c^* s = singlet; d = doublet; t = triplet.

### 2.2. Cytotoxicity Evaluation of **1**–**5**

Following their identification, compounds **1**–**5** were assessed for cytotoxicity in two high-grade ovarian cancer cell lines, OVCAR4 and Kuramochi [18]. Potential for selective cytotoxicity was evaluated using two non-cancerous mouse cell lines representing the putative progenitor cells of ovarian cancer, murine ovarian surface epithelial (MOSE) and murine oviductal epithelial (MOE). Screening of **1**–**5** against this panel revealed **3** and **4** to be non-specifically cytotoxic, which was in accord with their previously reported bioactivities [8,10,11,19,20,21]. Compound **2** exhibited up to 14-fold enhanced cytotoxicity against non-cancerous murine cell lines MOSE and MOE, with LC_50_ values of 9.80 µM and 10.8 µM, respectively. Generally speaking, this para-quinone scaffold is not an attractive lead, as higher toxicity in non-tumorigenic cell lines confers serious overall toxicity. In contrast, compound **5** showed approximately seven-fold greater activity toward Kuramochi ovarian cancer cells with an LC_50_ of 18.6 μM (Table 3). Previous studies reported that **5** inhibits tumor cell proliferation and viability in L1210 lymphocytic leukemia cells *in vitro* with an IC_50_ range of 0.24–0.65 µM [22]. Compound **1** was not significantly active in all bioassays in the current study.

**Table 3:** *In vitro* cytotoxicity of **1**–**5**.

Compound	Cytotoxicity LC_50_ (µM) *^a^*			
	Kuramochi	OVCAR4	MOSE	MOE
**1**	>33.1	>33.1	>33.1	>33.1
**2**	>32.5	>32.5	9.80	10.8
**3**	6.72	11.0	3.50	28.5
**4**	1.11	4.82	2.85	6.20
**5**	18.6	127	>149	>149

*^a^* Doxorubicin was used as the positive control and was lethal at the lowest concentration tested (0.078 µM).

Compounds **2**–**5** are classified as angucyclines, which are among the largest class of type II PKS natural products and are known to exhibit a wide variety of biological activities [23]. Compound **2** was initially isolated from marine *Streptomyces* sp. B8245 and reported in a dissertation, but it has yet to receive formal characterization [7]. Both **3** and **4** have previously been identified as intermediates in kinamycin biosynthesis [24,25]; this product was not detected in our fermentation extracts. Similarly, compound **3** was proven to be a biosynthetic intermediate of jadomycin and gilvocarcin in a *Streptomyces lividans* strain [26], though these were also not observed in secondary metabolite fractions produced by our *Micromonospora* strain. In previous reports, **3** exhibited no detectable activity when screened against a panel of Gram-negative and Gram-positive bacteria [27], but inhibited a wide range of cancer cell lines at micromolar potency. Compound **4** was reported to have a variety of antibiotic and cytotoxic properties and our data support these non-specific biological activities [10,11,21]. Finally, compound **5** was first isolated from a *Streptomyces auranticolor* strain and was reported to exhibit anticoccidial activity against the apicomplexan poultry parasite *Eimeria tenella*, while exhibiting no significant biological activity when screened against a panel that included two fungal species, a human parasite, and several Gram-negative and Gram-positive bacteria [12,13]. This compound was also studied more recently for its aforementioned antileukemic activity, where it inhibited proliferation leading to apoptosis through DNA cleavage, blockage of nucleoside transport and inhibition of DNA, RNA, and protein synthesis [22]. To our knowledge, this study is the first to report cytotoxicity of compound **5** in carcinomas. In most cases, **4** is significantly more cytotoxic than **3** and **5**. Presumably, this is due to the pyridine function in **4**. Additionally, a contributing factor to reduced cytotoxicity may be the loss of aromaticity in the adjacent anthraquinone ring (**5**), though further biological testing and structure activity analyses are required to support this.

## 3. Experimental Section 

### 3.1. General Experimental Procedures

Optical rotation measurement was performed in MeOH using a 10.0 cm cell on a PerkinElmer 241 polarimeter at 25 °C. The UV spectra were measured on a Shimadzu Pharma Spec UV-1700 spectrophotometer. NMR spectra were obtained on a Bruker 600 MHz DRX NMR spectrometer equipped with an inverse 5 mm TXI cryogenic probe with z-axis pfg and XWINNMR version 3.5 operating software, and a 900 (226.2) MHz Bruker AVANCE NMR spectrometer equipped with an inverse 5 mm TCI cryogenic probe with z-axis pfg and TopSpin version 1.3 operating software at the University of Illinois at Chicago Center for Structural Biology. Chemical shifts (δ) are given in ppm and coupling constants (*J*) are reported in Hz. ^1^H and ^13^C NMR resonances of **1** and **2** are reported in Table 1 and Table 2, respectively. ^1^H and ^13^C NMR chemical shifts were referenced to the CDCl_3_ (δ_H_ 7.26 ppm and δ_C_ 77.0 ppm, respectively) and C_6_D_6_ solvent signals (δ_H_ 7.16 ppm and δ_C_ 128.1 ppm, respectively). High resolution mass spectra were obtained on a Waters Synapt QToF mass spectrometer at the University of Illinois at Chicago Research Resources Center (UIC RRC). High-performance liquid chromatography (HPLC-UV) data were obtained using a Hewlett-Packard series 1100 system controller and pumps with a Model G1315A diode array detector (DAD) equipped with a reversed-phase C_18_ column (Phenomenex Luna, 100 mm × 4.6 mm, 5 μm; Torrance, CA, USA) at a flow rate of 0.5 mL·min^−1^. Semi-preparative HPLC scale separations were performed using a Hewlett Packard Series 1050 system with a Phenomenex Luna semi-preparative C_18_ column (250 mm × 10 mm, 5 μm) at a flow rate of 2.4 mL·min^−1^. Preparative HPLC scale separations were performed using a Waters LC4000 System equipped with a Phenomenex Luna preparative C_18_ column (250 mm × 21.2 mm, 5 μm) at a flow rate of 16 mL·min^−1^. Silica gel column chromatography was conducted using Bonna-Angela Technologies Cleanert^®^ silica gel (Wilmington, DE, USA) with an average particle size of 40–60 μm and an average pore size of 60 Å.

### 3.2. Collection and Identification of Actinomycete Strain G039

Strain G039 was isolated from a sediment sample collected by PONAR at a depth of 22 m, from *ca*. 3.3 miles off the coast southeast of Cát Bà Peninsula in Vietnam (20°41°30°′ N, 107°05°58°′ E) in July of 2011. Strain G039 (GenBank accession number KR703606) shared 99% 16S rRNA gene sequence identity with the type strain *Micromonospora haikouensis* (GenBank accession number NR117442) [28].

### 3.3. Fermentation and Extraction

Strain G039 was grown in 38 × 1 L portions in Fernbach flasks containing high nutrient media components in artificial seawater (10 g starch, 4 g yeast extract, 2 g peptone, 1 g calcium carbonate, 100 mg potassium bromide, 40 mg iron sulfate, and 33.3 g Instant Ocean^®^ (Blacksburg, VA， USA) per liter of dH_2_O) for 7 days at 21 °C while shaking at 220 rpm. Sterilized Amberlite XAD-16 resin (15 g·L^−1^) was added to each flask to absorb the extracellular secondary metabolites. The culture medium and resin were shaken for 10 h and filtered using cheesecloth to remove the resin. The resin, cell mass, and cheesecloth were extracted with acetone overnight, concentrated under vacuum, and partitioned between water and ethyl acetate. The organic layer was dried under vacuum to afford 3.52 g of extract.

### 3.4. Isolation and Characterization of Isopimara-2-one-3-ol-8,15-diene (**1**) and Lagumycin B (**2**)

The organic layer from the liquid-liquid partition was fractionated using silica gel flash column chromatography (23 cm × 3.5 cm, 85 g silica) eluting with a step gradient solvent system of 200 mL 100% hexanes (HEX), 200 mL 90% HEX:10% dichloromethane (DCM), 200 mL 70% HEX:30% DCM, 200 mL 100% DCM, 200 mL 70% DCM:30% ethyl acetate (EtOAc), 200 mL 70% EtOAc:30% DCM, 200 mL 100% ethyl acetate, 200 mL 95% EtOAc:5% 2-propanol, 200 mL 90% EtOAc:10% methanol (MeOH), 200 mL 50% EtOAc:50% MeOH, 400 mL 95% MeOH:5% ammonium hydroxide (NH_4_OH) to afford eleven fractions. Upon re-screening, it was determined that fraction 3 contained the bioactive constituents, thus, it was further separated using RP-C_18_ preparative HPLC (16 mL·min^−1^, gradient of 50% aqueous MeOH to 100% MeOH over 25 min, followed by an isocratic flow of 100% MeOH for 15 min), to afford 13 fractions. Fractions 6 and 11 exhibited biological activity so they were further separated.

Fraction 6 was further purified using RP-C_18_ semi-preparative HPLC (2.4 mL·min^−1^, gradient of 70% aqueous MeOH to 80% MeOH for 37.5 min, followed by an isocratic flow of 100% ACN for 10 min) to afford phenanthroviridone (**4**, *t*_R_ 37.5 min, 0.66 mg, 0.019% yield), lagumycin B (**2**, *t*_R_ 40.2 min, 0.9 mg, 0.026% yield) and WS-5995 A (**5**, *t*_R_ 42.0 min, 1.2 mg, 0.034% yield).

Fraction 11 was further separated using RP-C_18_ semi-preparative HPLC (2.4 mL·min^−1^, gradient of 80% aqueous MeOH to 90% MeOH for 37.5 min, followed by an isocratic flow of 100% ACN for 10 min) to afford 8 fractions. Fraction 2 was further purified using two iterations of RP-C_18_ semi-preparative HPLC (2.4 mL·min^−1^, gradient of 80% aqueous MeOH to 90% MeOH for 37.5 min, followed by an isocratic flow of 100% ACN for 10 min) to afford dehydrorabelomycin (**3**, *t*_R_ 26.7 min, 0.4 mg, 0.011% yield). Purification of compound **3** also afforded isopimara-2-one-3-ol-8,15-diene (**1**, *t*_R_ 27.5 min, 0.9 mg, 0.026% yield), eluting as a neighboring peak observable at 220 nm.

Isopimara-2-one-3-ol-8,15-diene (**1**): Colorless solid (0.9 mg). ${\left[\text{α}\right]}_{\text{D}}^{25}$ = +20.7 (*c =* 0.00053, MeOH). UV (MeOH) λ_max_ (log ε) = 204 (3.67) and shoulders at 228 (3.39) and 242 (3.30) nm. CD (*c* = 0.0031, MeOH): λ_max_ (Δε) = 225 (+65.2), 250 (−4.4), 290 (+54.4) nm (Figure S11). ^1^H NMR (900 MHz, CDCl_3_) and ^13^C NMR (226.2 MHz, CDCl_3_), see Table 1. HRESI-QToF MS *m*/*z* 303.2338 [M + H]^+^ (calcd. for C_20_H_31_O_2_: 303.2319), and *m*/*z* 325.2141 [M + Na]^+^ (calcd. for C_20_H_30_O_2_Na: 325.2138).

Lagumycin B (**2**): Pale orange solid (0.9 mg). UV (MeOH) λ_max_ (log ε) = broad absorptions with maxima at 204 (3.57), 280 (2.86), 310 (2.73) and 410 (2.27) nm. ^1^H NMR (600 MHz, CDCl3) and ^13^C NMR (226.2 MHz, CDCl_3_), see Table 2. HRESI-QToF MS *m*/*z* 309.0773 [M + H]^+^ (calcd. for C_18_H_13_O_5_: 309.0757).

### 3.5. OVCAR4, Kuramochi, MOSE, MOE, and Vero Cytotoxicity Assays

Human ovarian OVCAR4 and Kuramochi cancer cells were maintained in RPMI 1640 (11875-093, Life-technologies; Carlsbad, CA, USA) supplemented with 10% FBS (16000-044, Life-technologies; Carlsbad, CA, USA) and 1% penicillin/streptomycin. Non-cancerous murine ovarian surface epithelium (MOSE) and murine oviductal epithelium (MOE) cells were maintained as previously reported [29]. Concentration-response experiments were performed as previously described for 72 h [6].

## 4. Conclusions

Of the five compounds isolated in this study, two were previously unreported. Compound **1** represents a relatively rare example of the isolation of a diterpene from an actinomycete strain. Since a previous report that mined the genomes of a small population of actinomycete strains suggested that this phylum has a far greater capacity to produce diterpenes (25 of 100 strains) than is represented in the peer reviewed literature, it is possible that this discrepancy is a product of these pathways remaining silent under laboratory growth conditions, or of the methods used to isolate natural products from bacteria, namely bioassay-guided fractionation [3]. One possible explanation of the latter phenomenon is that specific classes of diterpenes are conserved within Actinobacteria, and that these classes are not biologically active in the typical barrage of assays used to discover small molecule leads, though in the absence of extensive genome mining and subsequent structure identification-bioactivity experiments, this will remain speculation.

The novel diterpene isopimara-2-one-3-ol-8,15-diene (**1**) was isolated along with the angucycline lagumycin B (**2**), and three other angucyclines from a *Micromonospora* sp. collected in the East Sea of Vietnam. The angucyclines exhibited varying degrees of cytotoxicity against a panel of cancerous and non-cancerous cell lines, with the notable exception of WS-5995 A (**5**), which showed enhanced cytotoxicity against the Kuramochi cell line when compared to non-tumorigenic cell lines. Though not significantly active in our bioassays, the discovery of isopimara-2-one-3-ol-8,15-diene contributes to the growing but still relatively small number of diterpenes identified from actinomycetes. This study highlights our collaborative efforts to discover novel biologically active molecules from the large, underexplored, and biodiversity-rich waters of Vietnam’s East Sea.

## Supplementary Files

Supplementary File 1
