# Supplementary material for: A Pimarane Diterpene and Cytotoxic Angucyclines from a Marine-Derived Micromonospora sp. in Vietnam’s East Sea
Source: Mar Drugs. 2015 Sep 15;13(9):5815–27. doi: 10.3390/md13095815 (PMC4584356; doi:10.3390/md13095815)
Supplement: Supplementary File 1 [file marinedrugs-13-05815-s001.docx]

Supplementary Materials

*In vitro* Evaluation of 1–5 in *M. tuberculosis* Whole Cell Assays

The microplate alamar blue assay (MABA) was used to test the ability of compounds **1**–**5** to inhibit replicating *M. tuberculosis* H37Rv ATCC 27294 *in vitro* as previously described [1–3]. Compounds **2**–**4** exhibited minimum inhibitory concentrations of 2.01 μg/mL, 1.01 μg/mL, and 0.72 μg/mL (MICs; defined as the lowest concentration resulting in ≥90% growth inhibition of H37Rv, and averaged from triplicates), respectively, while compounds **1** and **5** lacked significant activity. Additionally, none of the compounds were significantly active when tested for their ability to inhibit non-replicating *M. tuberculosis* H37Rv ATCC 27294 in the low oxygen recovery assay (LORA) as previously described (Table S1) [1–4]. Given the cytotoxic nature of compounds **2**–**4**, the antibiotic activity observed here is not specific to bacteria.

**Table S1.** *In vitro* anti-*M. tuberculosis* activity of **1**–**5**.

| **Compound** | **H_37_Rv MIC (μg/mL)** | |
| --- | --- | --- |
|  | **MABA *^a^*** | **LORA *^a^*** |
| **1** | 19.4 | >50 |
| **2** | 2.01 | 25.7 |
| **3** | 1.01 | 19.6 |
| **4** | 0.72 | 17.8 |
| **5** | 22.7 | >50 |

*^a^* MABA and LORA assays were performed by Baojie Wan and Sanghyun Cho at the Institute for Tuberculosis Research at UIC.

**Table S2.** ^1^H NMR data (600 MHz) of **1** in CDCl3 and C6D6.

| **Position** | **^1^H, Mult. (*J*, Hz) in CDCl_3_ *^a^*** | | **^1^H, Mult. (*J*, Hz) in C_6_D_6_ *^a^*** |
| --- | --- | --- | --- |
| 1_ax_ | 2.25, d (12.3) | | 1.76, d (12.3) |
| 1_eq_ | 2.58, d (12.3) | | 2.40, d (12.3) |
| 2 |  | |  |
| 3 | 3.91, d (4.0) | | 3.59, d (3.7) |
| 3-OH | 3.44, d (4.0) | | 3.66, d (3.7) |
| 4 |  | |  |
| 5 | 1.78, m | | 1.45, dd (2.0, 12.5) |
| 6 | 1.58, m | | 1.31, m |
|  | 1.80, m | 1.50, m | |
| 7 | 2.03, m | | 1.76, m |
| 8 |  | |  |
| 9 |  | |  |
| 10 |  | |  |
| 11_ax_ | 1.76, m | | 1.58, m |
| 11_eq_ | 1.88, m | | 1.77, m |
| 12_eq_ | 1.33, m | | 1.15, m |
| 12_ax_ | 1.53, m | | 1.38, m |
| 13 |  | |  |

**Table 2.** *Cont.*

| 14 | 1.76, m | | 1.60, m |
| --- | --- | --- | --- |
|  | 1.88, m | 1.77, m | |
| 15 | 5.72, dd (17.5, 10.7) | | 5.73, dd (17.5, 10.7) |
| 16 | 4.85, dd (17.5, 1.4) | | 4.91, dd (17.5, 1.4) |
|  | 4.92, dd (10.7, 1.4) | 4.99, dd (10.7, 1.4) | |
| 17 | 0.98, s | | 0.98, s |
| 18 | 0.93, s | | 0.81, s |
| 19 | 1.21, s | | 1.13, s |
| 20 | 0.72, s | | 0.72, s |

*^a^* s = singlet; d = doublet; dd = doublet of doublets; m = multiplet.


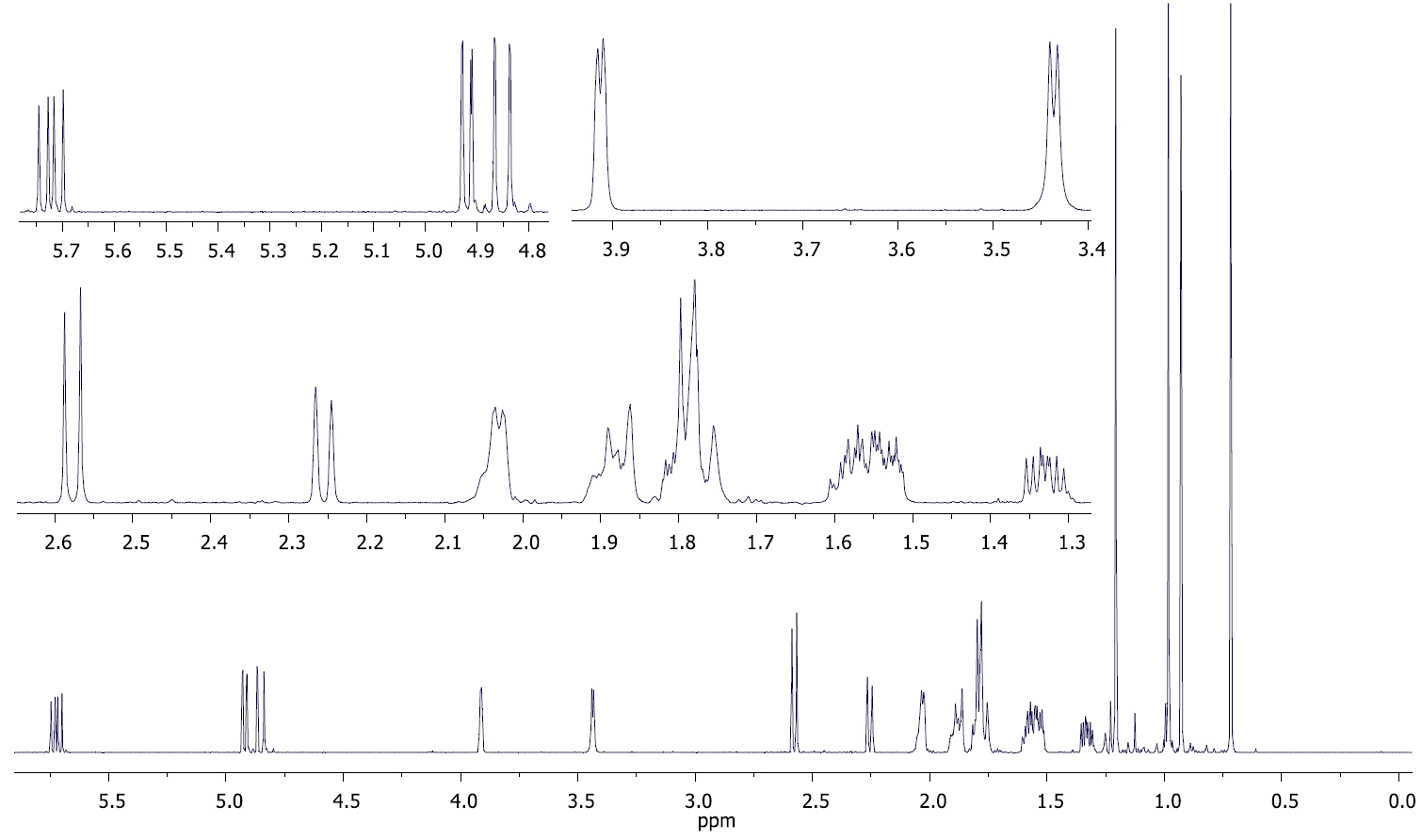


**Figure S1.** ^1^H NMR spectrum (600 MHz) of **1** in CDCl3.


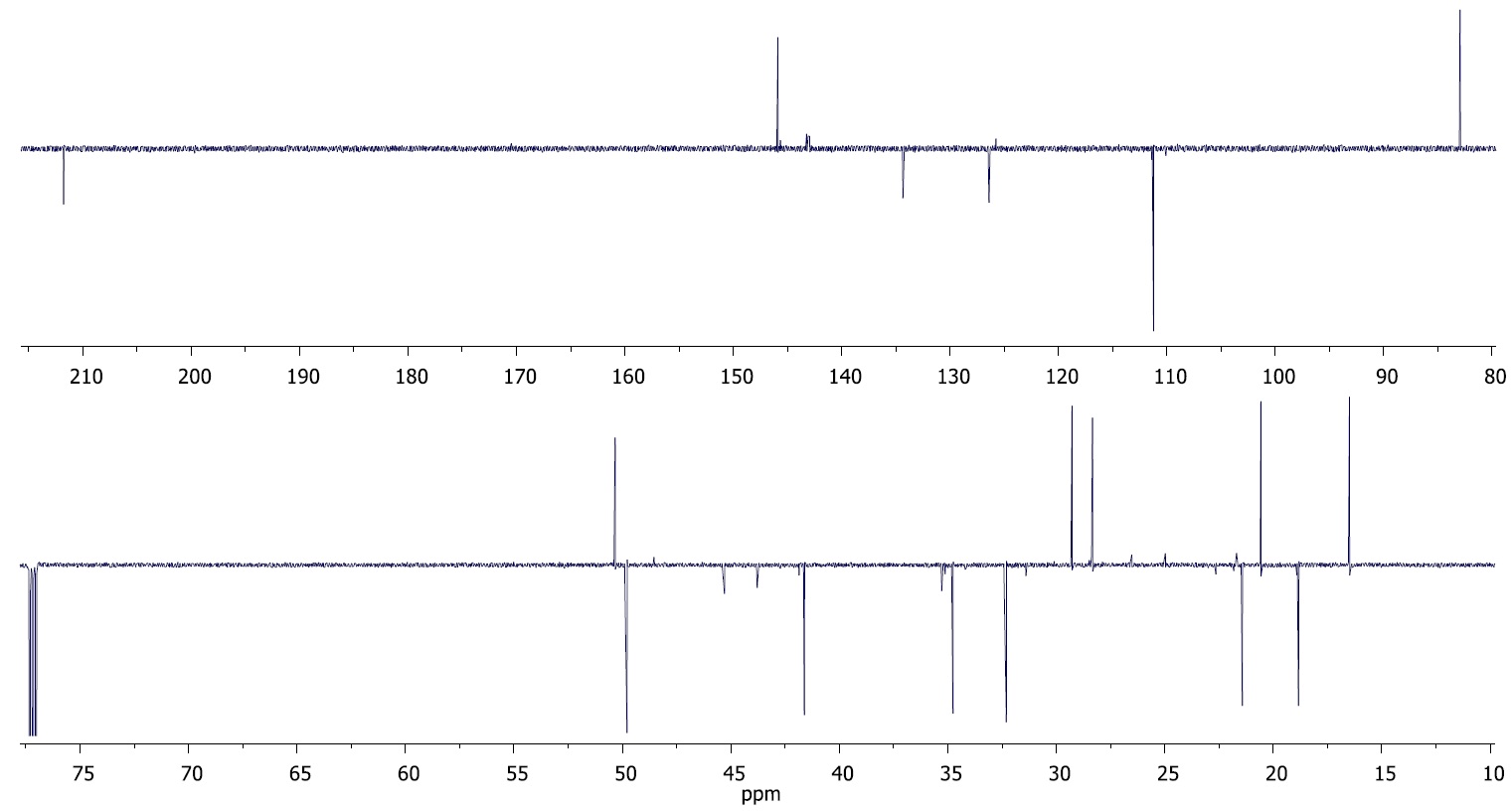


**Figure S2.** ^13^C DEPTQ spectrum (226.2 MHz) of **1** in CDCl3.


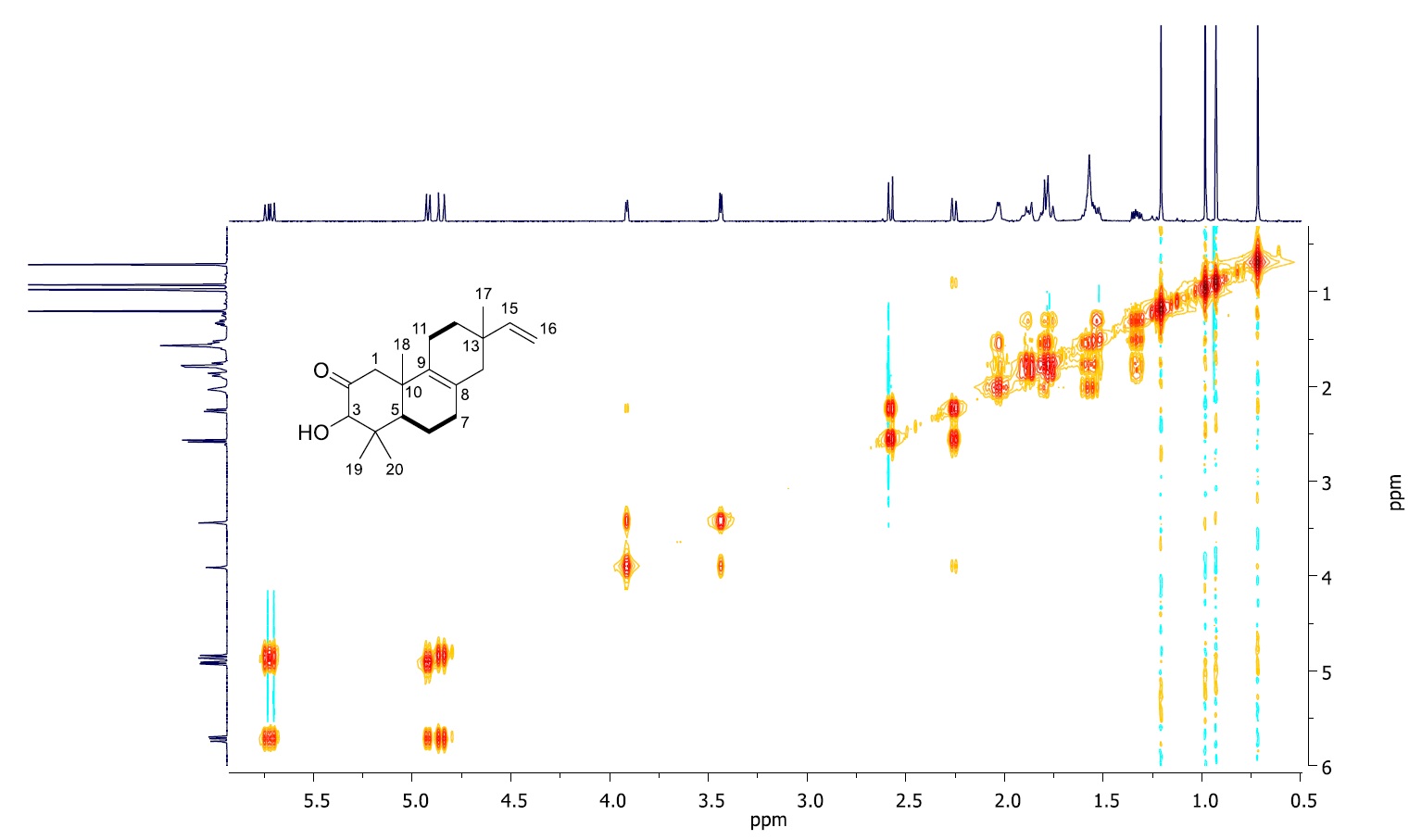


**Figure S3.** COSY spectrum (600 MHz) of **1** in CDCl3.


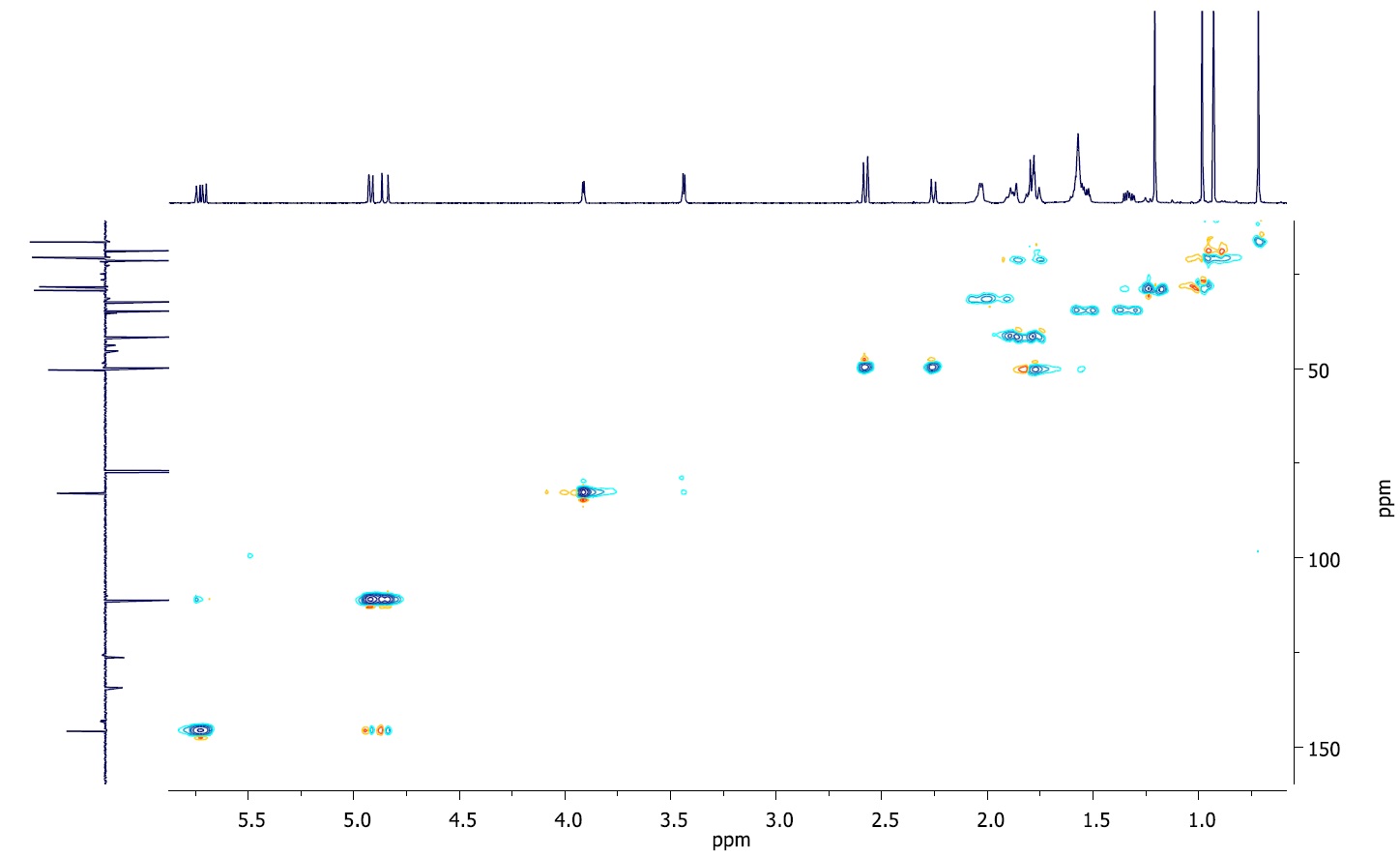


**Figure S4.** HSQC spectrum (600 MHz) of **1** in CDCl3.


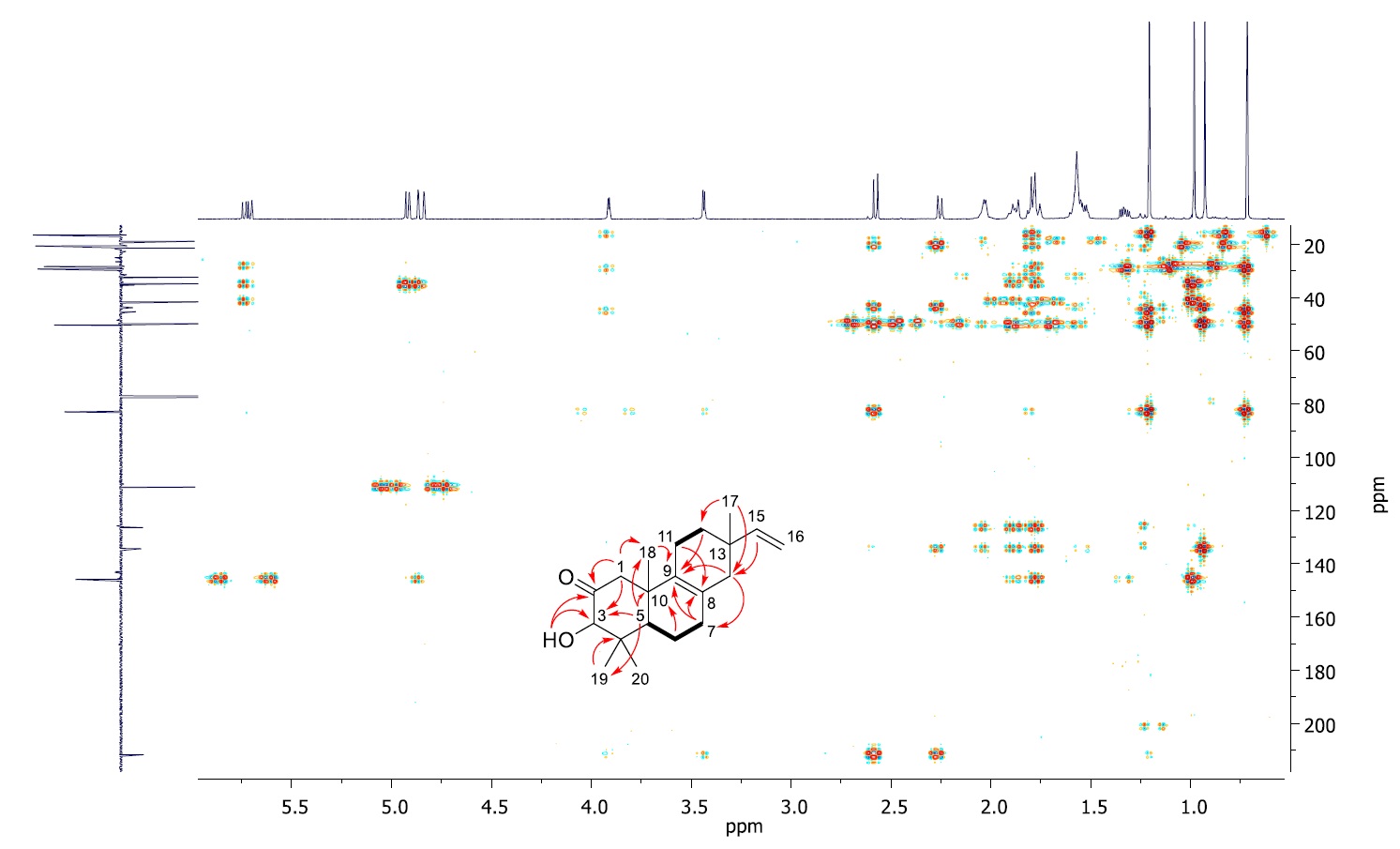


**Figure S5.** HMBC spectrum (600 MHz) of **1** in CDCl3.


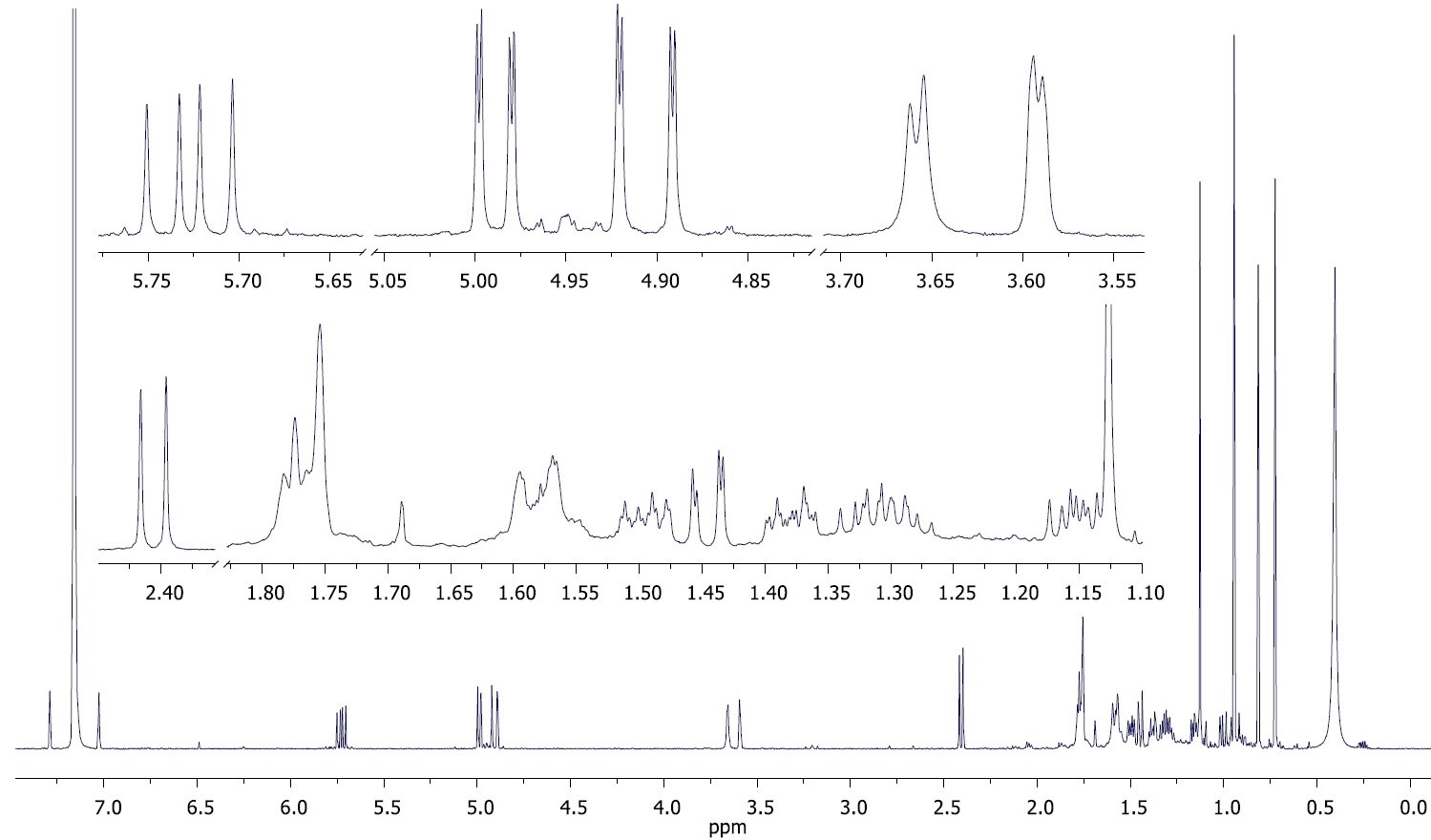


**Figure S6.** ^1^H NMR spectrum (600 MHz) of **1** in C6D6.


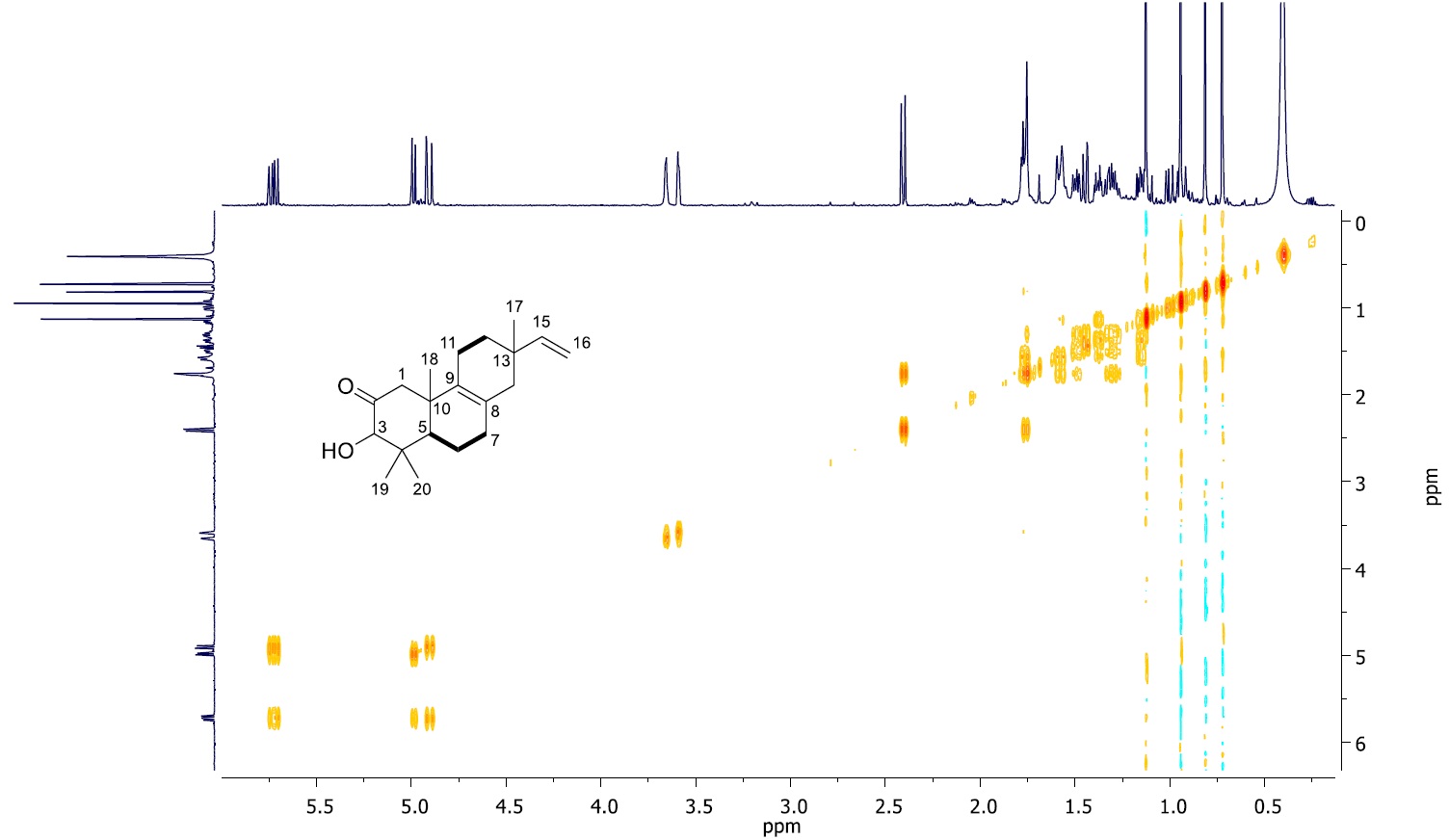


**Figure S7.** COSY spectrum (600 MHz) of **1** in C6D6.


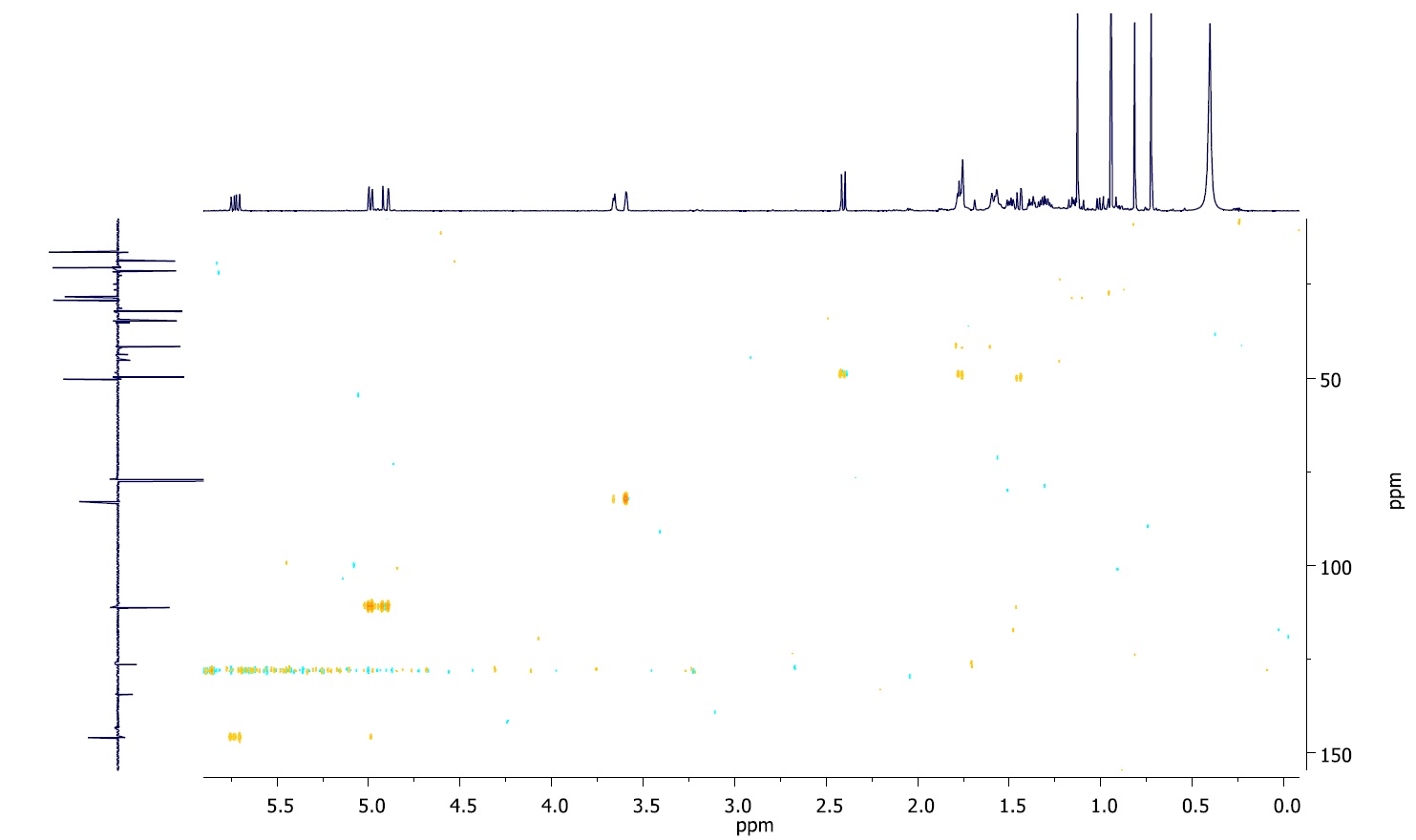


**Figure S8.** HSQC spectrum (600 MHz) of **1** in C6D6.


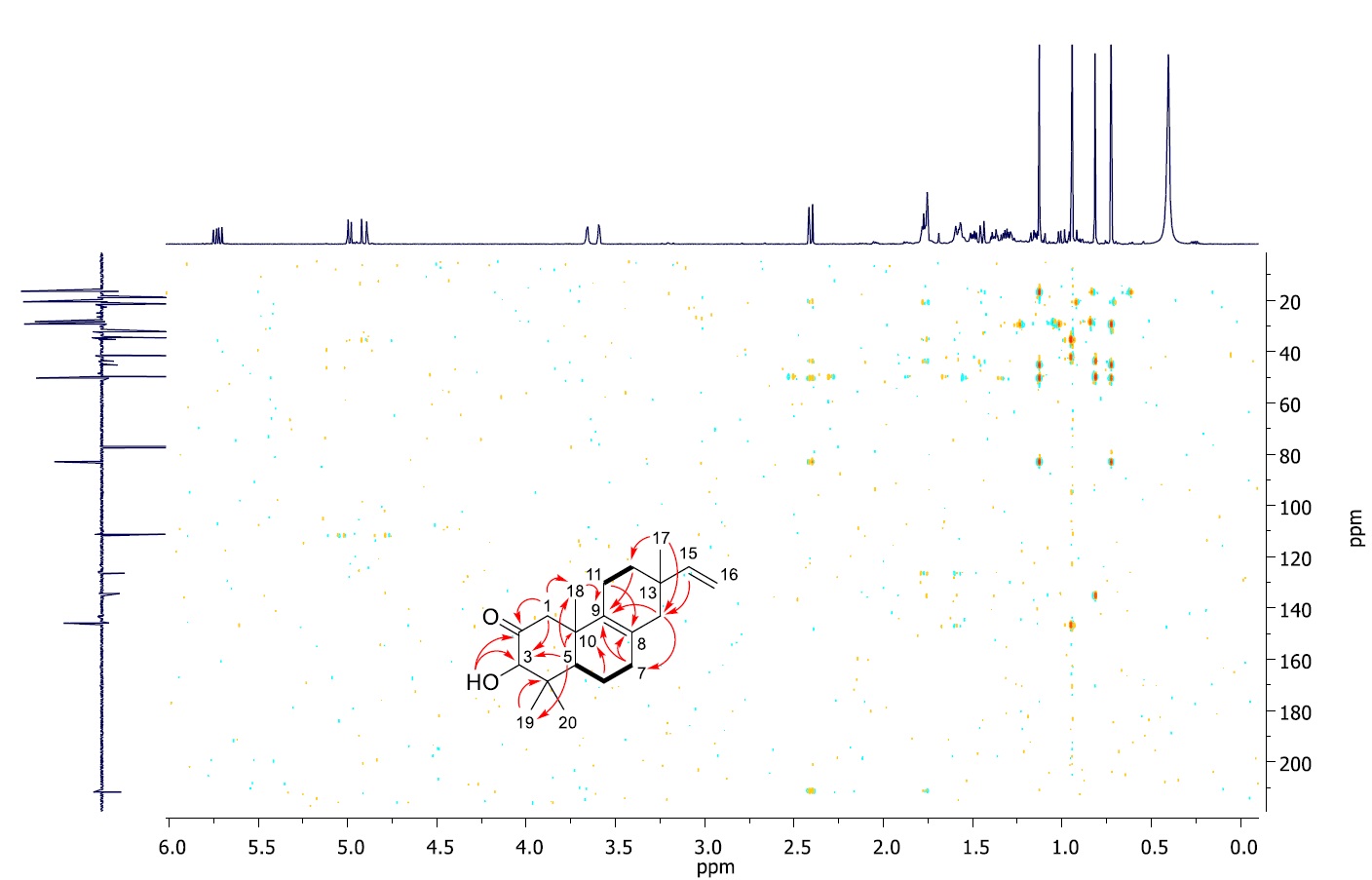


**Figure S9.** HMBC spectrum (600 MHz) of **1** in C6D6.


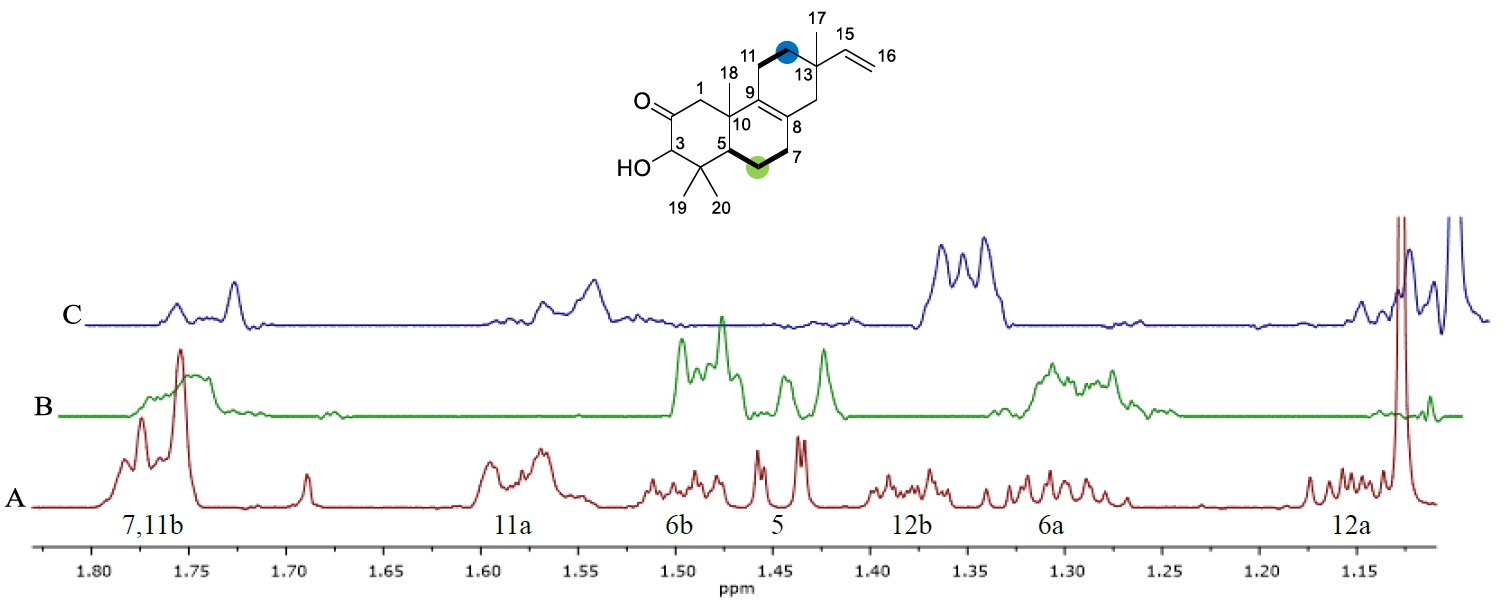


**Figure S10.** 1D-TOCSY spectra (600 MHz) of **1** in C6D6. (**A**) Expansion of ^1^H NMR spectrum (600 MHz) of **1**; (**B**) Expansion of 1D TOCSY spectrum of **1** (irradiation of δH 1.30); (**C**) Expansion of 1D TOCSY spectrum of **1** (irradiation of δH 1.15).


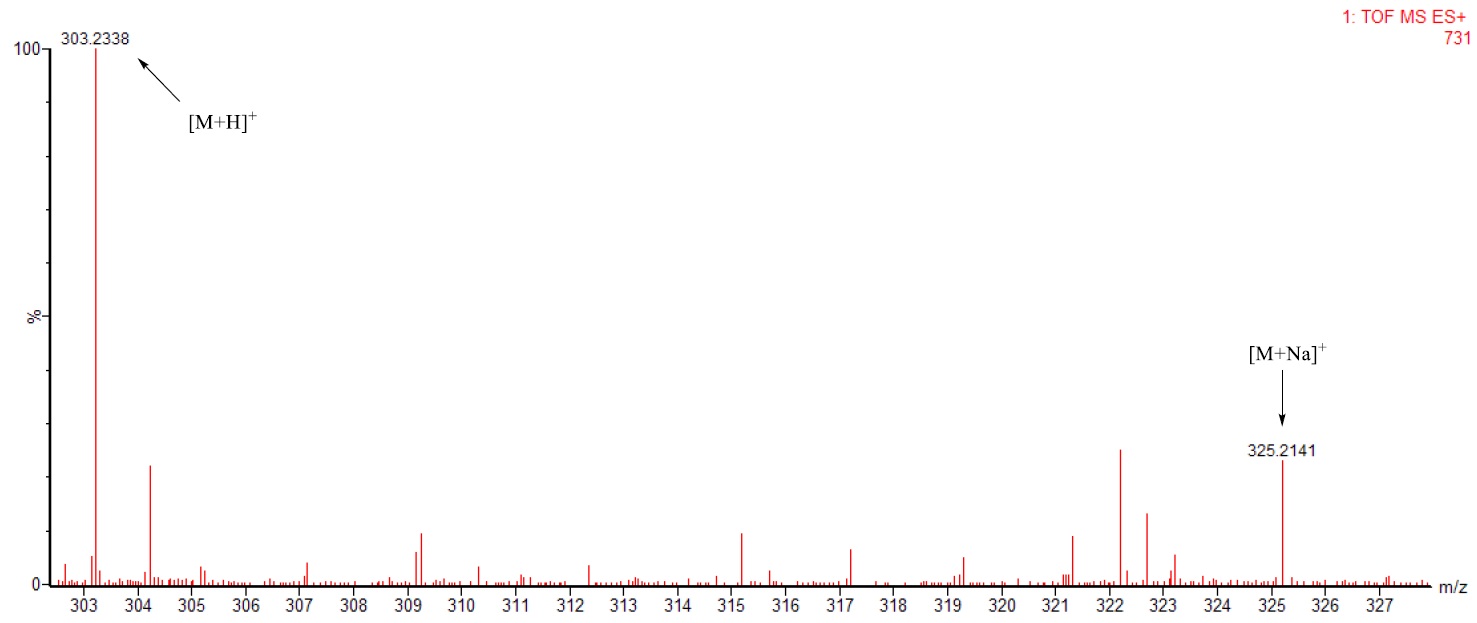


**Figure S11.** Expanded HR-ESI-QToF mass spectrum of **1**. HR-ESI-QToF MS *m*/*z* 303.2338 [M + H]^+^ (calcd. for C20H31O2: 303.2319), and *m*/*z* 325.2141 [M + Na]+ (calcd. for C20H30O2Na: 325.2138).


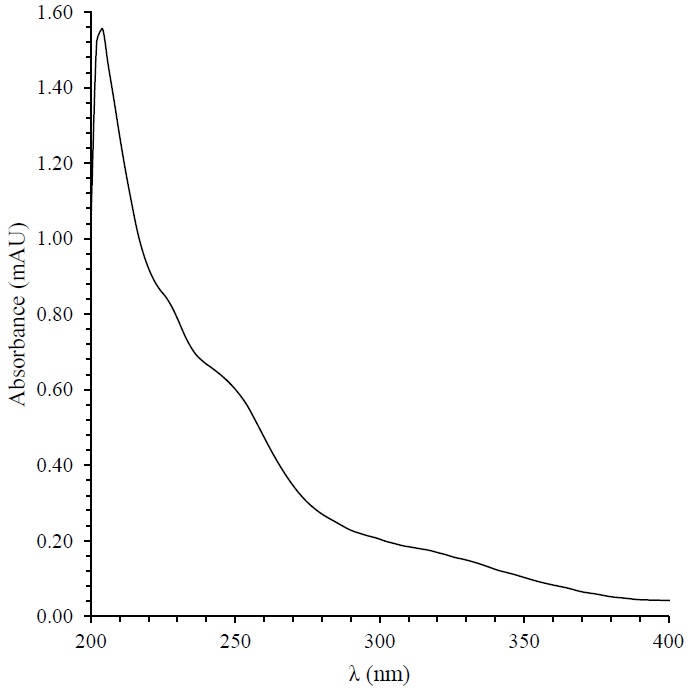


**Figure S12.** UV spectrum of **1** in methanol. UV (MeOH) λmax (log ε) = 204 (3.67) and shoulders at 228 (3.39) and 242 (3.30) nm.


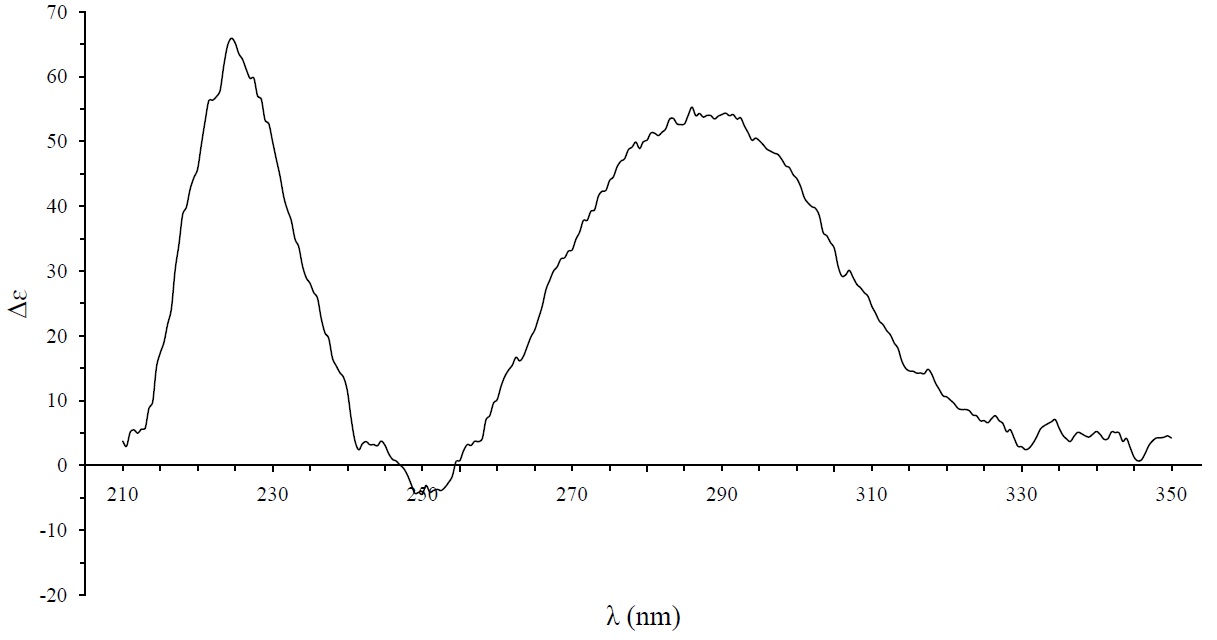


**Figure S13.** CD spectrum of **1** in methanol. CD (*c* = 0.0031, MeOH): λmax (Δε) = 225 (+65.2), 250 (−4.4), 290 (+54.4) nm.


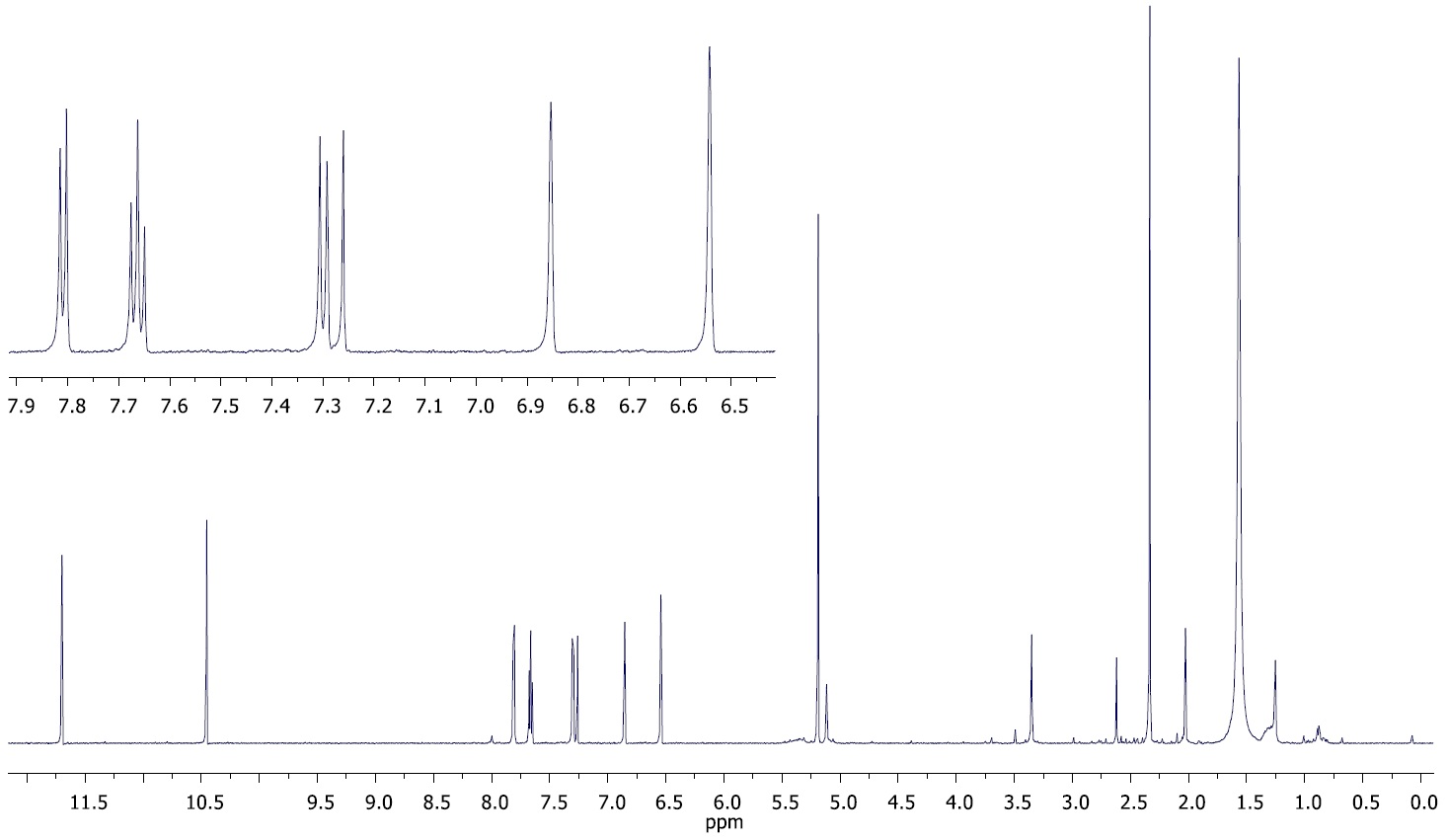


**Figure S14.** ^1^H NMR spectrum (600 MHz) of **2** in CDCl3.


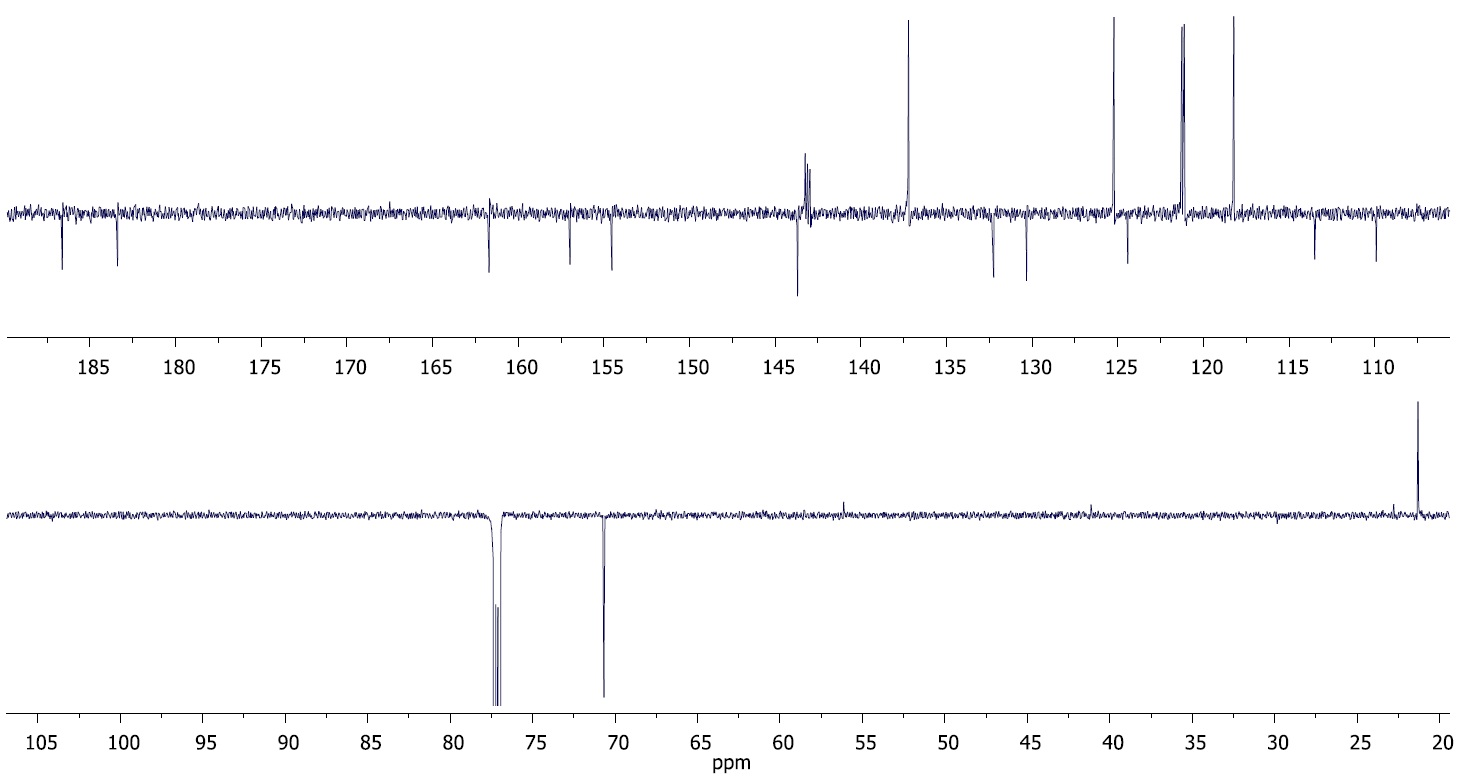


**Figure S15.** ^13^C DEPTQ spectrum (226.2 MHz) of **2** in CDCl3.


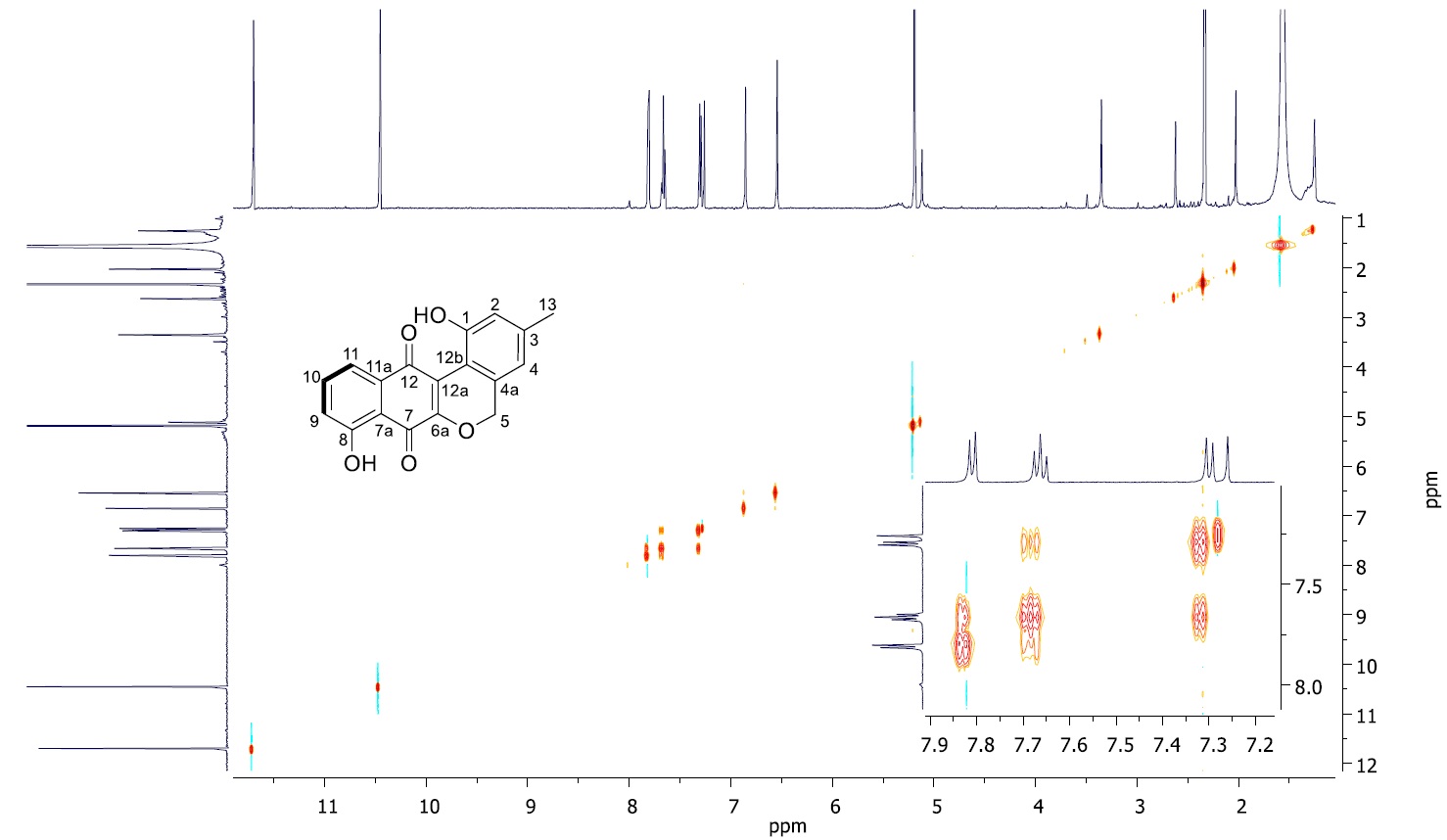


**Figure S16.** COSY spectrum (600 MHz) of **2** in CDCl3.


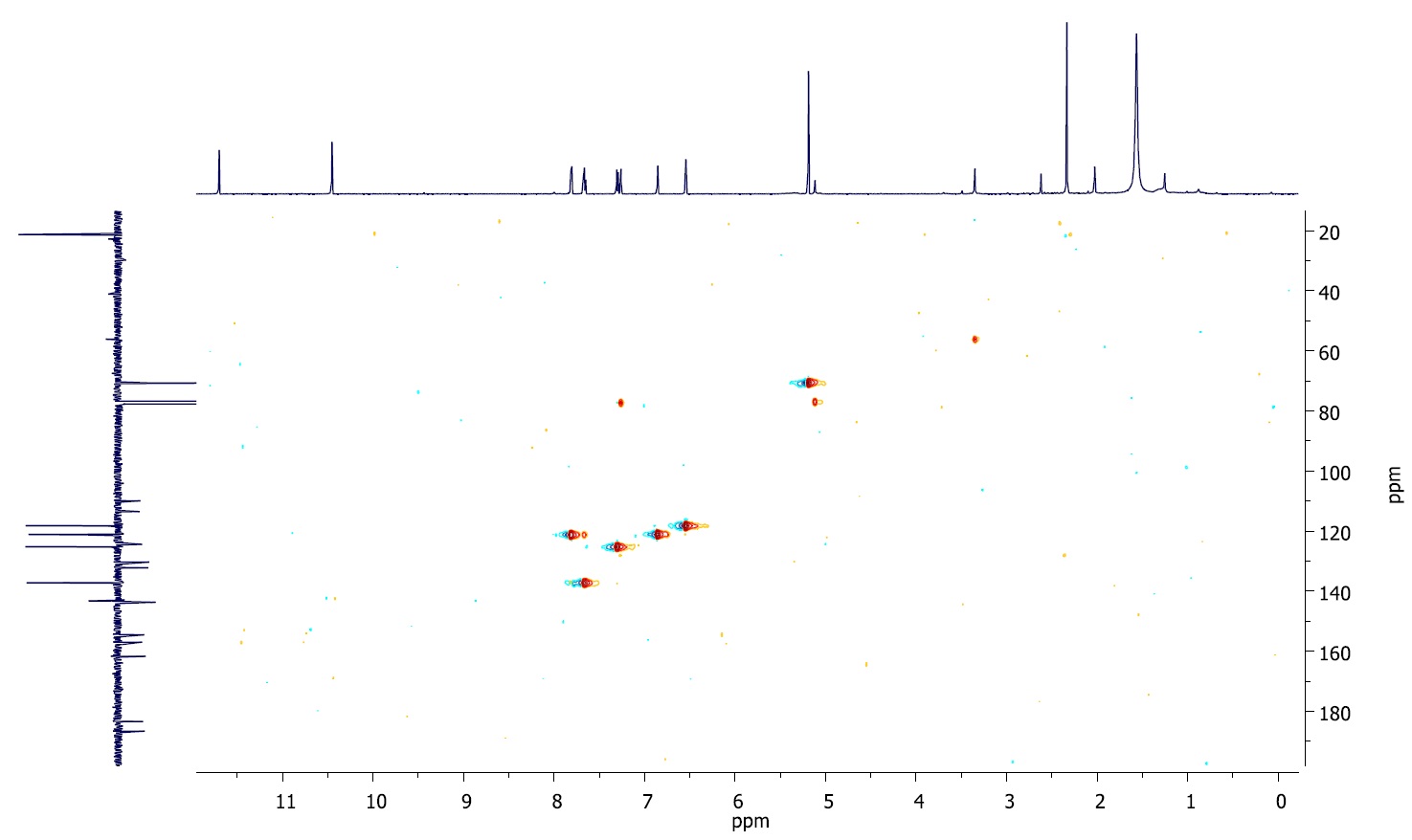


**Figure S17.** HSQC spectrum (600 MHz) of **2** in CDCl3.


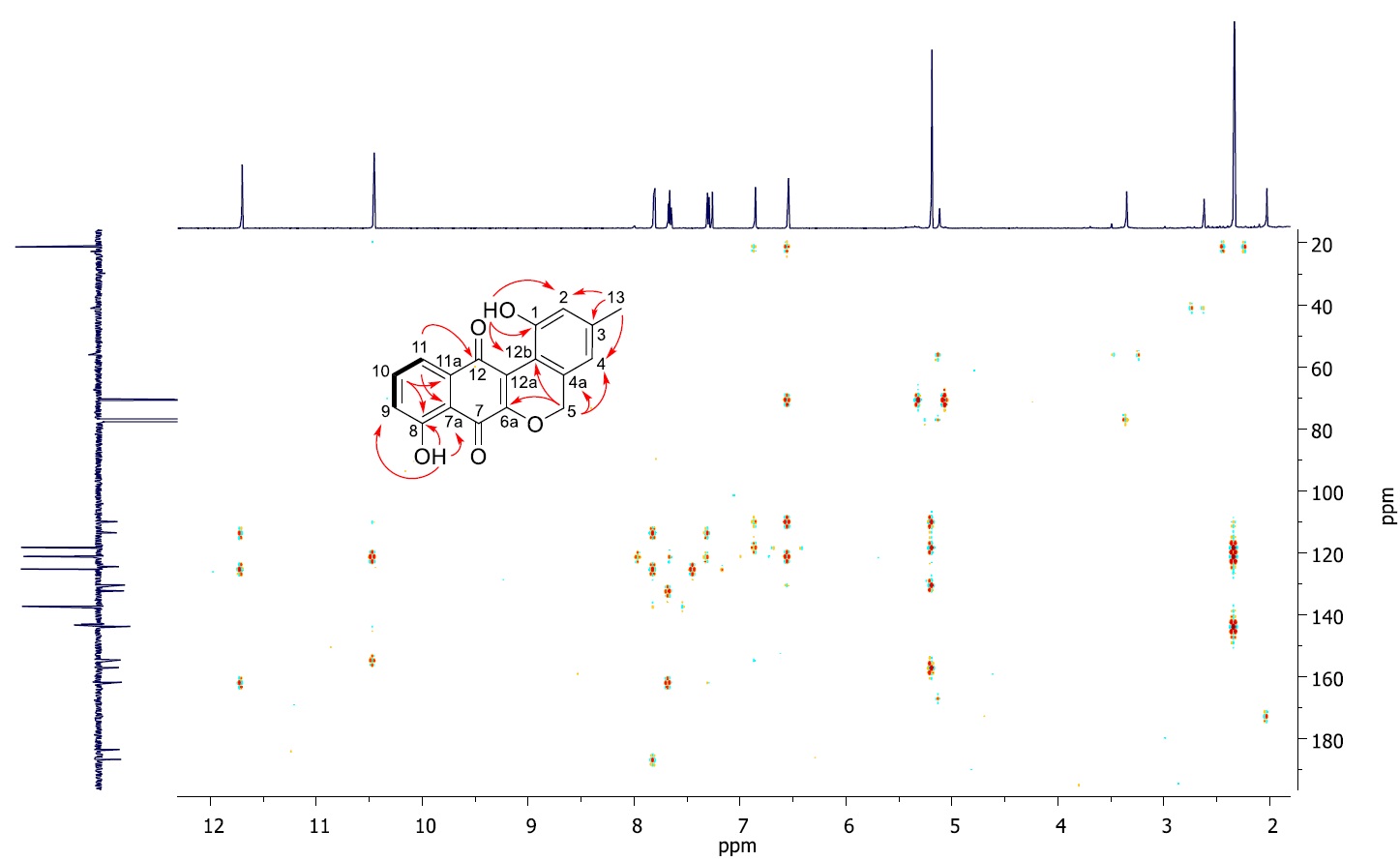


**Figure S18.** HMBC spectrum (600 MHz) of **2** in CDCl3.


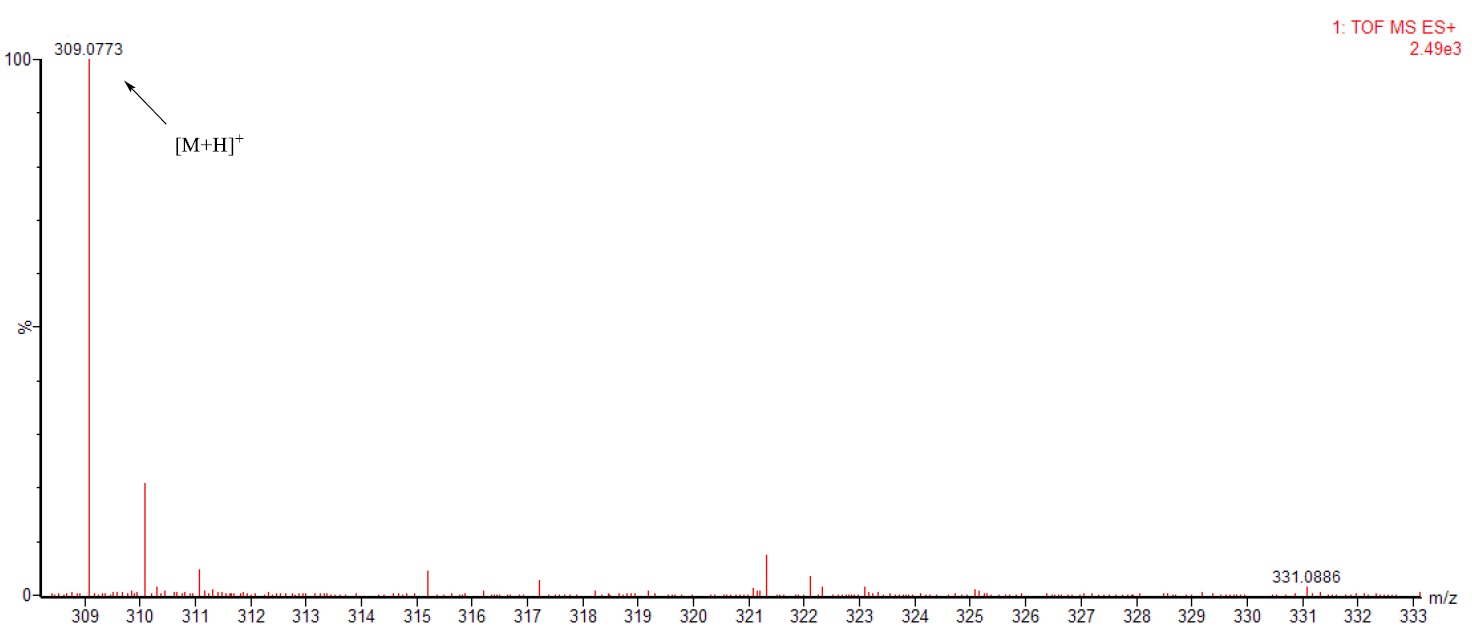


**Figure S19.** Expanded HR-ESI-QToF mass spectrum of **2**. HR-ESI-QToF MS *m*/*z* 309.0773 [M + H]^+^ (calcd. for C18H13O5: 309.0757).


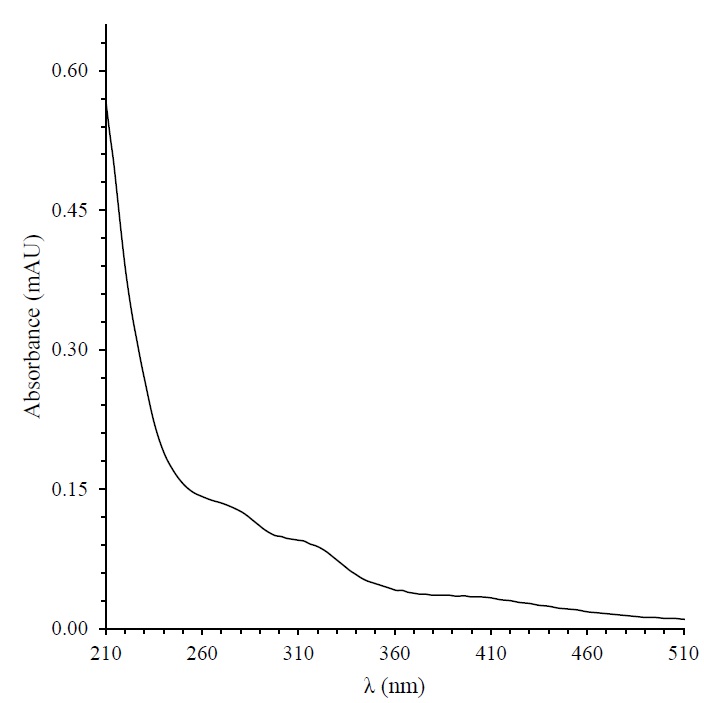


**Figure S20.** UV spectrum of **2** in methanol. UV (MeOH) λmax (log ε) = broad absorptions with maxima at 204 (3.57), 280 (2.86), 310 (2.73) and 410 (2.27) nm.


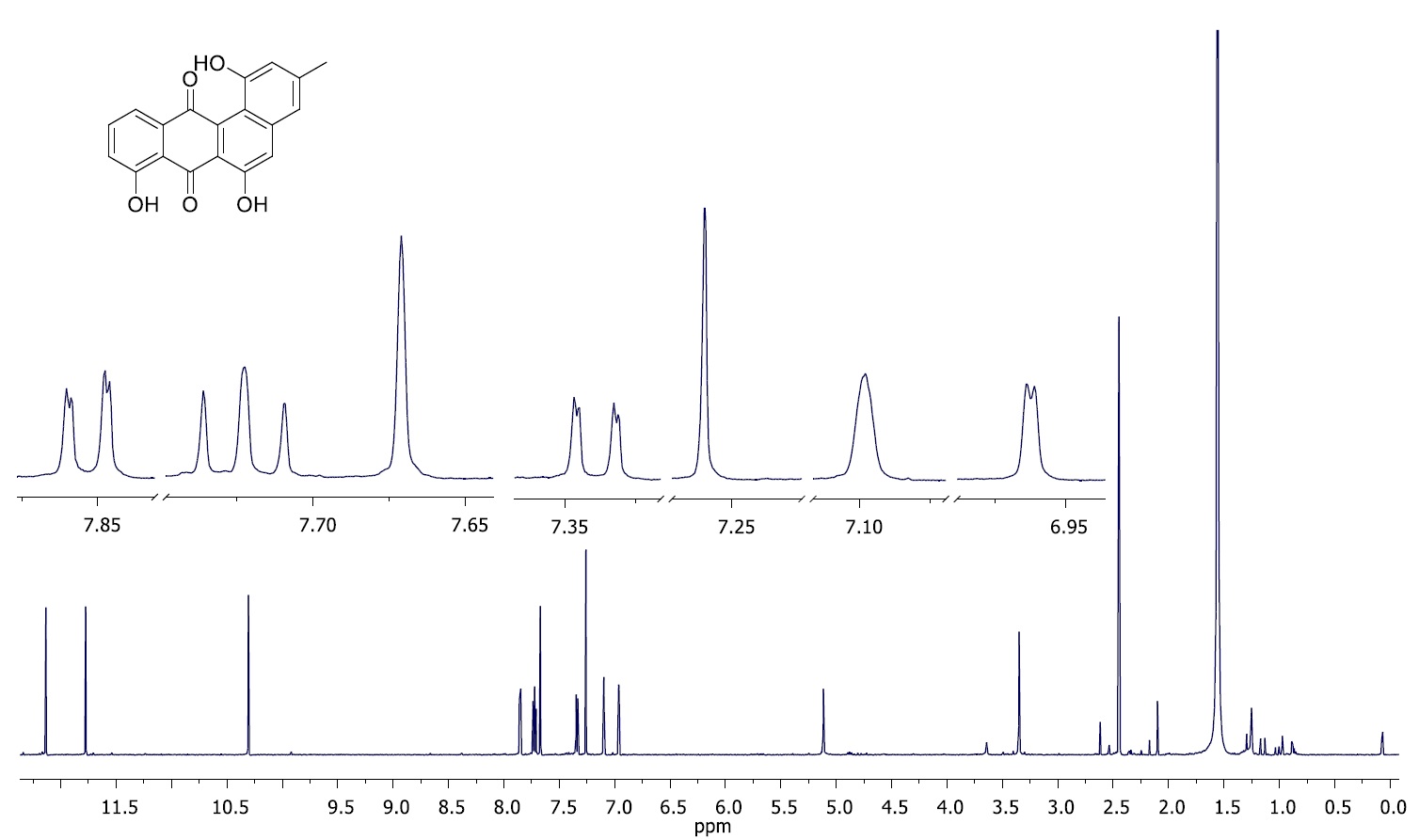


**Figure S21.** ^1^H NMR spectrum (600 MHz) of dehydrorabelomycin (aka: 6-hydroxytetrangulol, **3**) [5–6] in CDCl_3_.


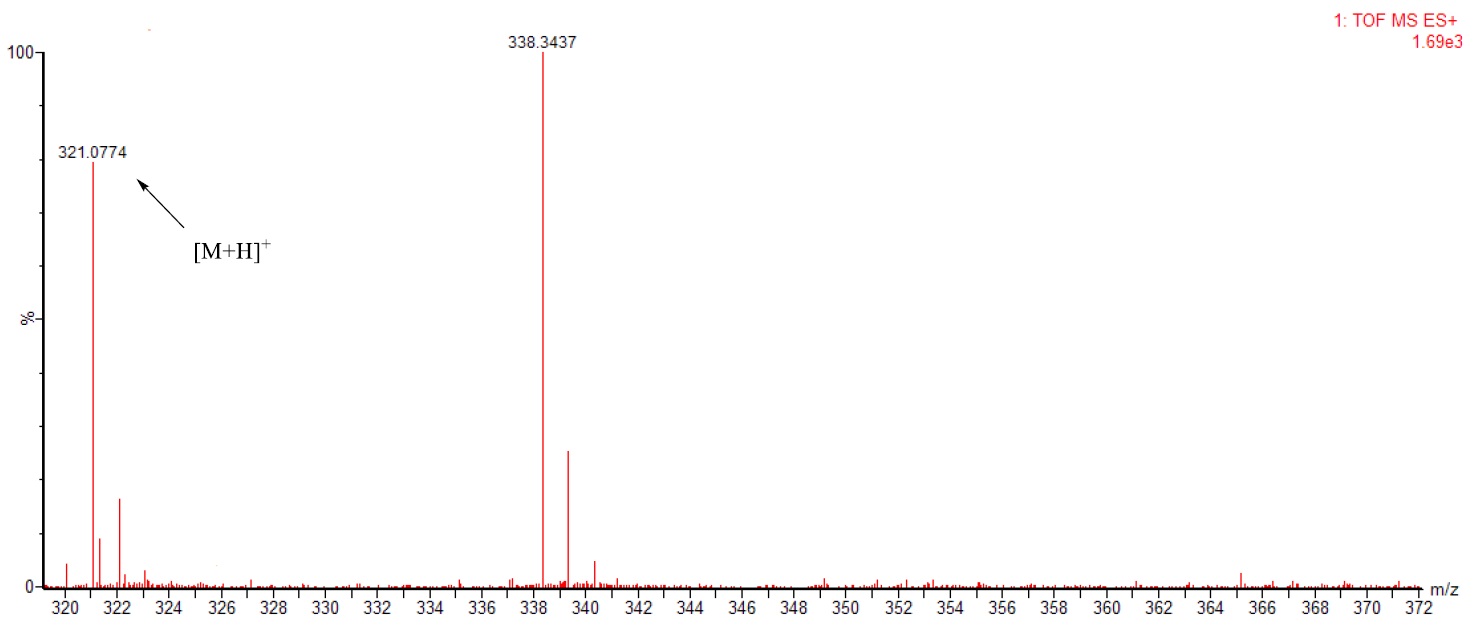


**Figure S22.** Expanded HR-ESI-QToF mass spectrum of **3**. HR-ESI-QToF MS *m*/*z* 321.0763 [M + H]^+^ (calcd. for C19H13O5: 321.0757).


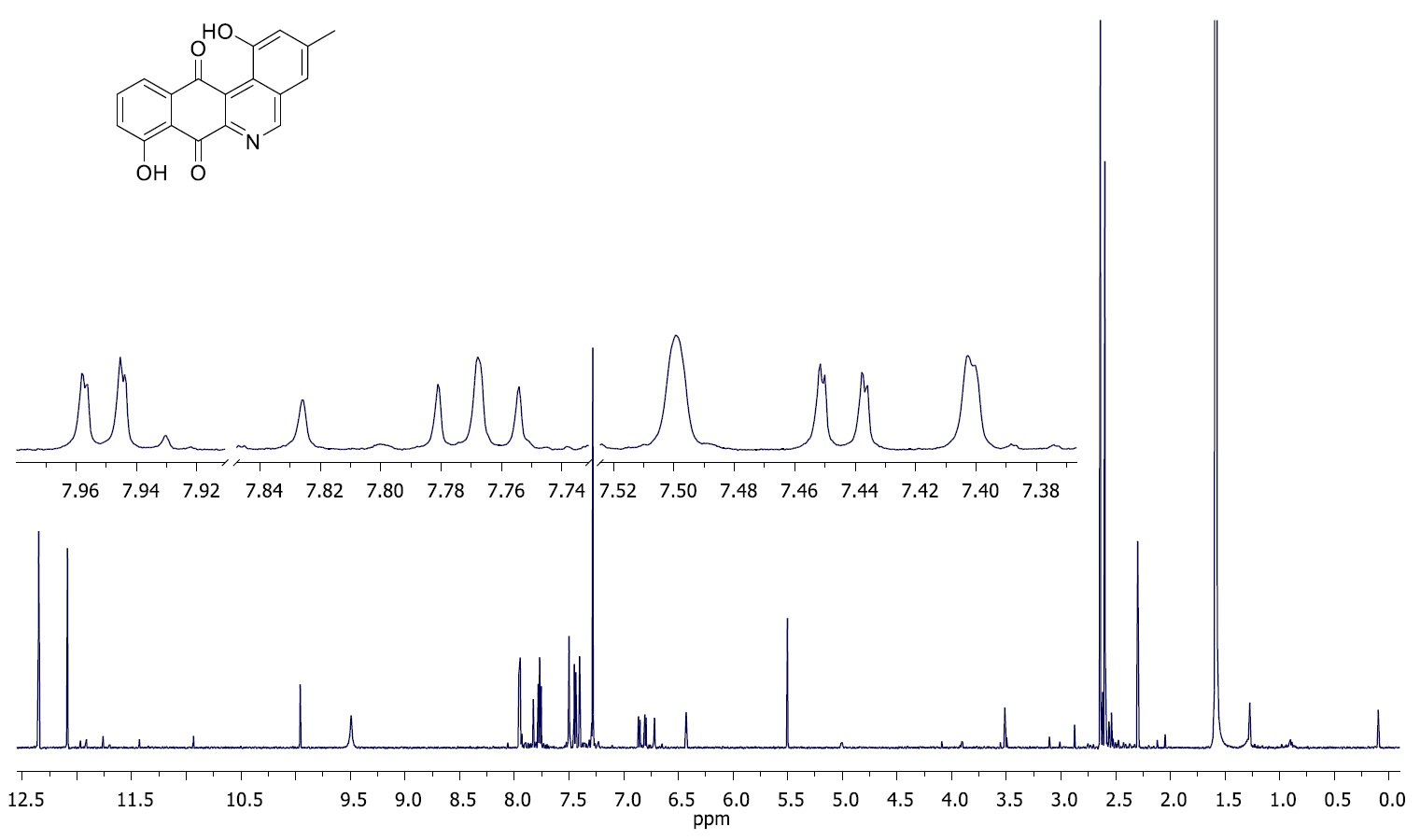


**Figure S23.** ^1^H NMR spectrum (600 MHz) of phenanthroviridone (aka: phenanthroviridin aglycone, **4**) [7–8] in CDCl3.


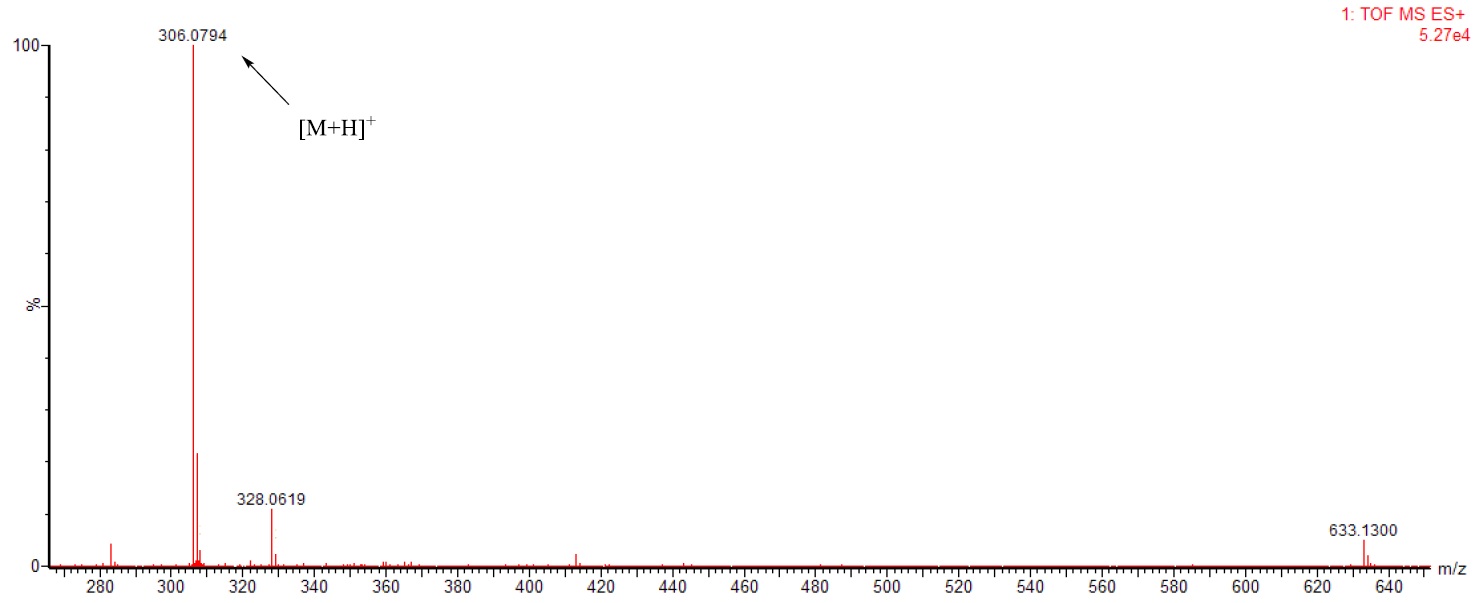


**Figure S24.** Expanded HR-ESI-QToF mass spectrum of **4**. HR-ESI-QToF MS *m*/*z* 306.0794 [M + H]^+^ (calcd. for C18H12NO4: 306.0761).


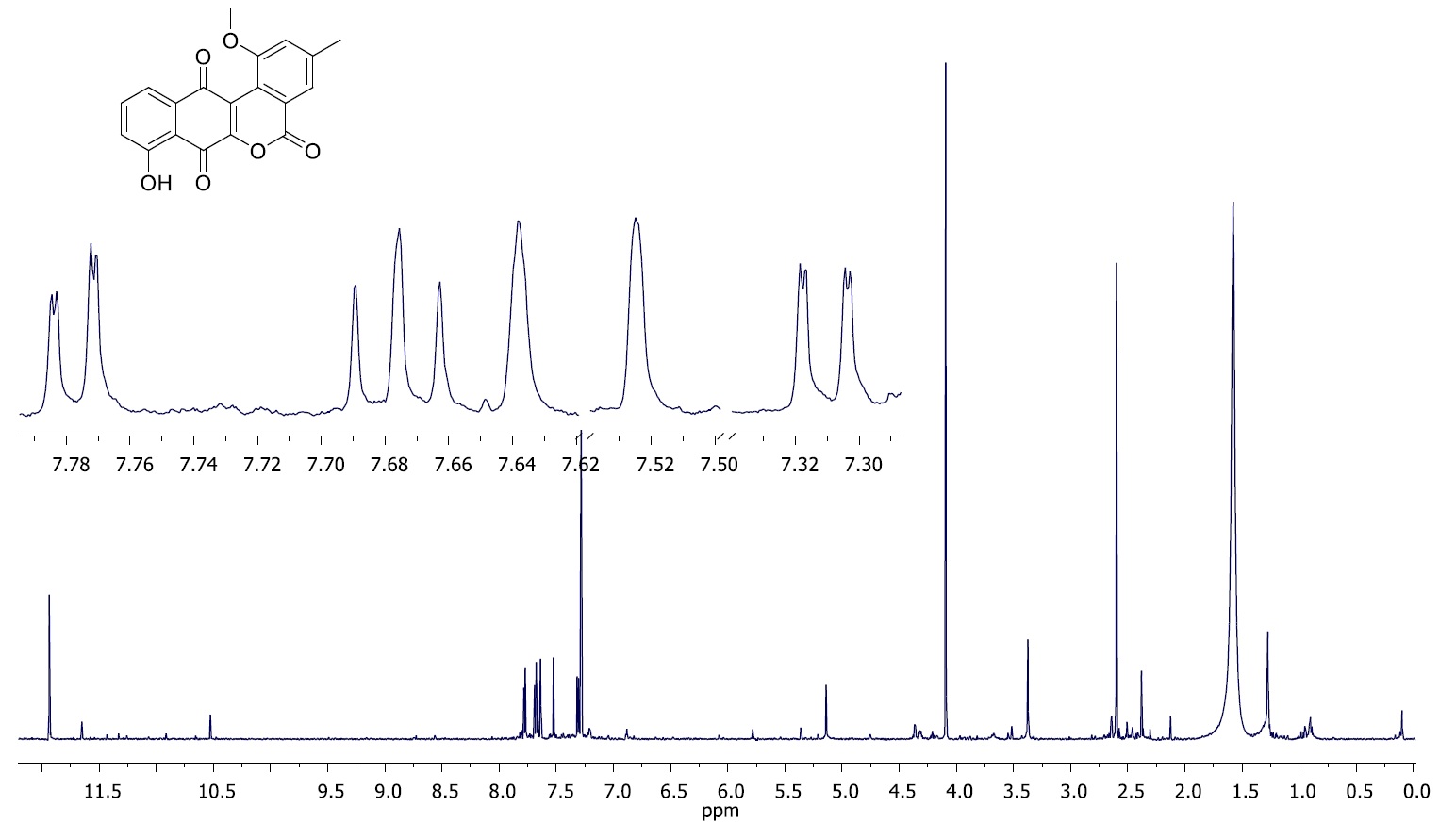


**Figure S25.** ^1^H NMR spectrum (600 MHz) of WS-5995 A (**5**) [9–10] in CDCl3.


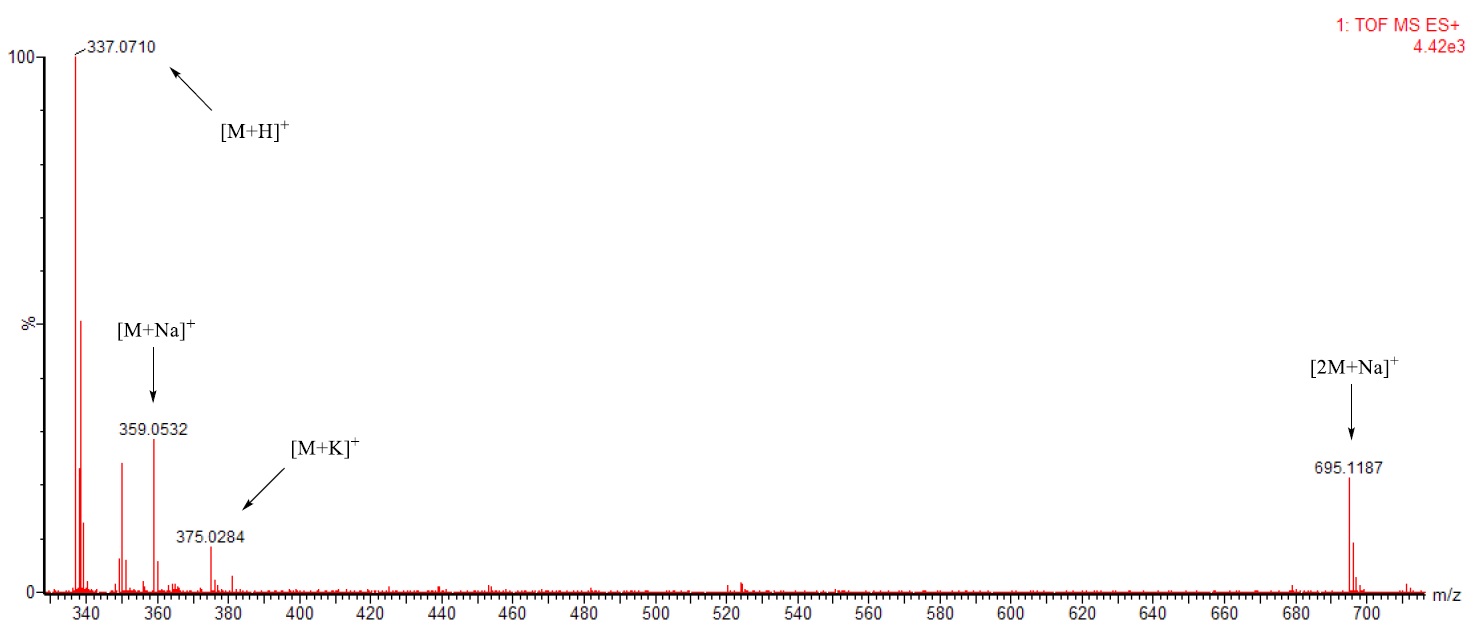


**Figure S26.** Expanded HR-ESI-QToF mass spectrum of **5**. HR-ESI-QToF MS *m*/*z* 337.0710 [M + H]^+^ (calcd. for C19H13O6: 337.0707), *m*/*z* 359.0532 [M + Na]^+^ (calcd. for C19H12O6Na: 359.0526), *m*/*z* 375.0284 [M + K]^+^ (calcd. for C19H12O6K: 375.0265), and *m*/*z* 695.1187
[2M + Na]^+^ (calcd. for C38H24O12Na: 695.1160).

References

1. Mullowney, M.W.; Hwang, C.H.; Newsome, A.G.; Wei, X.; Tanouye, U.; Wan, B.; Carlson, S.; Barranis, N.J.; Ó hAinmhire, E.; Chen, W.-L.; *et al*. Diaza-anthracene Antibiotics from a
   Freshwater-Derived Actinomycete with Selective Antibacterial Activity toward *Mycobacterium tuberculosis*. *ACS* *Infect. Dis.* **2015**, *1*, 168–174.
2. Collins, L.; Franzblau, S.G. Microplate alamar blue assay versus BACTEC 460 system for
   high-throughput screening of compounds against *Mycobacterium tuberculosis* and *Mycobacterium avium*. *Antimicrob.* *Agents Chemother.* **1997**, *41*, 1004–1009.
3. Franzblau, S.G.; Witzig, R.S.; McLaughlin, J.C.; Torres, P.; Madico, G.; Hernandez, A.; Degnan, M.T.; Cook, M.B.; Quenzer, V.K.; *et al.* Rapid, low-technology MIC determination with clinical *Mycobacterium tuberculosis* isolates by using the microplate Alamar Blue assay. *J. Clin. Microbiol.* **1998**, *36*, 362–366.
4. Cho, S.H.; Warit, S.; Wan, B.; Hwang, C.H.; Pauli, G.F.; Franzblau, S.G. Low-oxygen-recovery assay for high-throughput screening of compounds against nonreplicating *Mycobacterium tuberculosis*. *Antimicrob. Agents. Chemother.* **2007**, *51*, 1380–1385.
5. Yamashita, N.; Takashi, H.; Kazuo, S.; Haruo, S. 6-Hydroxytetrangulol, a new CPP32 protease inducer produced by *Streptomyces* sp. *J. Antibiot.* **1998**, *1*, 79–81.
6. Liu, W.C.; Parker, L.; Slusarchyk, S.; Greenwood, G.L.; Grahm, S.F.; Meyers, E. Isolation, characterization, and structure of rabelomycin, a new antibiotic. *J. Antibiot.* **1970**, *23*, 437–441.
7. Fendrich, G.; Zimmermann, W.; Gruner, J.; Auden, J.A.L. Phenanthridine Derivatives, Process for the Preparation Thereof, and Compositions Containing Them. U.S. Patent 5,093,247, 3 March 1990.
8. Gore, M.P.; Gould, S.J.; Weller, D.D. Synthesis of putative intermediates in the biosynthesis of the kinamycin antibiotics: Total synthesis of phenanthroviridin aglycon and related compounds. *J. Org. Chem.* **1992**, *57*, 2774–2783.
9. Ikushima, H.; Iguchi, E.; Kohsaka, M.; Aoki, H.; Imanaka, H. *Streptomyces auranticolor* sp. nov., a new anticoccidial antibiotics producer. *J. Antibiot.* **1980**, *33*, 1103–1106.
10. Ikushima, H.; Okamoto, M.; Tanaka, H.; Ohe, O.; Kohsaka, M.; Aoki, H.; Imanaka, H. New anticoccidial antibiotics, WS-5995 A and B: I. Isolation and characterization. *J. Antibiot.* **1980**, *33*, 1107–1113.

© 2015 by the authors; licensee MDPI, Basel, Switzerland. This article is an open access article distributed under the terms and conditions of the Creative Commons Attribution license (http://creativecommons.org/licenses/by/4.0/).
